# Genomic Variations in the Structural Proteins of SARS-CoV-2 and Their Deleterious Impact on Pathogenesis: A Comparative Genomics Approach

**DOI:** 10.3389/fcimb.2021.765039

**Published:** 2021-10-13

**Authors:** Taj Mohammad, Arunabh Choudhury, Insan Habib, Purva Asrani, Yash Mathur, Mohd Umair, Farah Anjum, Alaa Shafie, Dharmendra Kumar Yadav, Md. Imtaiyaz Hassan

**Affiliations:** ^1^ Centre for Interdisciplinary Research in Basic Sciences, Jamia Millia Islamia, New Delhi, India; ^2^ Department of Computer Science, Jamia Millia Islamia, New Delhi, India; ^3^ Department of Microbiology, University of Delhi, New Delhi, India; ^4^ Department of Clinical Laboratory Sciences, College of Applied Medical Sciences, Taif University, Taif, Saudi Arabia; ^5^ Department of Pharmacy and Gachon Institute of Pharmaceutical Science, College of Pharmacy, Gachon University, Incheon, South Korea

**Keywords:** severe acute respiratory syndrome coronavirus-2, coronavirus disease 2019, single amino acid substitutions, SARS-CoV-2 mutations, SARS-CoV-2 pathogenesis

## Abstract

A continual rise in severe acute respiratory syndrome coronavirus-2 (SARS-CoV-2) infection causing coronavirus disease (COVID-19) has become a global threat. The main problem comes when SARS-CoV-2 gets mutated with the rising infection and becomes more lethal for humankind than ever. Mutations in the structural proteins of SARS-CoV-2, i.e., the spike surface glycoprotein (S), envelope (E), membrane (M) and nucleocapsid (N), and replication machinery enzymes, i.e., main protease (M^pro^) and RNA-dependent RNA polymerase (RdRp) creating more complexities towards pathogenesis and the available COVID-19 therapeutic strategies. This study analyzes how a minimal variation in these enzymes, especially in S protein at the genomic/proteomic level, affects pathogenesis. The structural variations are discussed in light of the failure of small molecule development in COVID-19 therapeutic strategies. We have performed in-depth sequence- and structure-based analyses of these proteins to get deeper insights into the mechanism of pathogenesis, structure-function relationships, and development of modern therapeutic approaches. Structural and functional consequences of the selected mutations on these proteins and their association with SARS-CoV-2 virulency and human health are discussed in detail in the light of our comparative genomics analysis.

## Introduction

Severe acute respiratory syndrome coronavirus-2 (SARS-CoV-2), a seventh strain and the third member of the coronavirus family, has rapidly spread all across the globe since 2019 and has been a leading cause of death worldwide (Seo et al., 2020). The urgency and health crisis forced the World Health Organization (WHO) to enforce a state of health emergency and declare it a pandemic (Jebril, 2020). People with existing comorbidity and those belonging to the elderly were more prone to this infection earlier. Still, now many young individuals are losing the battle to Coronavirus disease 2019 (COVID-19) (Ruan, 2020; Brookman et al., 2021). Such changes in the patterns of SARS-CoV-2 infection as compared to the previous strains of coronaviruses and among different variants of SARS-CoV-2 has been attributed to the mutations of the virus in the Spike (S) protein, a part of the structural component which allows it to enter into the host cells (Wan et al., 2020). Currently, the total number of confirmed cases across the world as of now stands at > 219 million, whereas 4.5 million people have died (assessed on 11^th^ September 2021) (Organization, 2020). Many countries have witnessed this expeditious spread of infection in the form of different waves occurring after a certain time interval (Asrani et al., 2021c; Boroujeni et al., 2021; Iftimie et al., 2021). Many scientists have warned against the upcoming peaks of the current waves and the arrival of new waves, which are yet to come in several countries in the future (Lai and Cheong, 2020). The incubation period of this virus is said to be between 10-14 days. Therefore, immediate diagnosis post-viral entry is not possible, putting undue pressure on the healthcare infrastructure and medical facilities. Diagnosis is mostly performed through reverse transcription real-time – polymerase chain reaction (rRT-PCR) approach, but it takes time to provide results (Singh et al., 2020a; Singh et al., 2020b; Vo et al., 2020; Asrani et al., 2021a). Rapid antigen test is also being used in certain parts; however, rRT-PCR is a more accurate procedure to be followed for the diagnosis (Asrani et al., 2021b). The serious complications of this virus have led to the complete lockdown in major parts of the world, leading to physical and psychological effects on their citizens (Ju et al., 2021; Kim et al., 2021; Van Vo et al., 2021).

SARS-CoV-2 mediates its entry into the host *via* the S protein of the virus, which interacts with the ACE2 receptors on the host cells (Lan et al., 2020). In this positive sense, a single-stranded RNA virus escapes the host’s innate and adaptive immune response, causing overproduction of cytokines leading to the formation of cytokine storm (Song et al., 2020). Patients in serious conditions have shown an alleviated expression of IL-2, IL-7, IL-10, IP10, MIP1A, MCP1, G-CSF and TNFα cytokines (Huang C. et al., 2020). The death is mainly observed to be caused by pneumonia affecting the patient’s respiratory system (Xu et al., 2020). Along with acute respiratory distress syndrome, COVID-19 causes the manifestation of acute heart injuries, heart failures, inflammation leading to sepsis and multi-organ dysfunction in individuals in chronic cases (Wang D. et al., 2020). The virus was initially thought to spread through droplets of infected individuals *via* sneezing or coughing; however, recent reports claim their airborne transmission (Zou et al., 2020; Tang et al., 2021).

The virus possesses four structural proteins- spike (S) protein that helps in attachment of the virus to the host cells ACE-2 receptors (Kandeel et al., 2018); membrane (M) protein typically involved in the formation of viral membrane for enclosing the mature virus particles (Neuman et al., 2011); nucleocapsid (N) protein involved in the formation of a viral protein coat, i.e., N which surrounds the genetic material of the virus (Risco et al., 1996); and envelope (E) protein which is involved in the formation of the envelope that assembles the virion particles (Ruch and Machamer, 2012). The following gene arrangement has been observed in SARS-CoV-2 structural analysis: 5' untranslated region (UTR) [non-structural genes (ORF 1a/ORF1b replicase gene), structural genes (S, M, E, and N) and accessory genes (ORF 3, ORF 6, ORF 7a, ORF 7b, ORF 8, ORF 9b)] 3' UTR (Song et al., 2019; Asrani et al., 2020). Replicase genes account for the synthesis of non-structural proteins (NSPs). Sixteen NSPs assist in the replication and packaging of the virus (Naqvi et al., 2020). Accessory proteins usually differ among the different strains of Coronaviruses (Li et al., 2020). SARS-CoV-2 shares more than 80% genomic similarity to the previous SARS-CoV strain that caused an outbreak in 2003 (Asrani et al., 2020; Malik, 2020). Thus, it is known to exhibit a similar replication process as observed in the previous cases.

Now, different mutant strains of this virus have been identified from different parts of the world, such as B.1.1.7 variant of SARS-CoV-2 was originally acknowledged in United Kingdom (UK), B.1.351 variant from South Africa, B.1.1.28 variant from Brazil, B.1.36 variant, N440K and E484Q mutations from India however; all these variants have now been identified and cultured following their spread to different parts of the world (Tang J. W. et al., 2020; Islam et al., 2021; Planas et al., 2021). Apart from these single-site mutations, few variants have been reported to have double and triple mutations. B.1.617, a double mutant variant that originated from a combination of previously identified Coronavirus variants L452R and E484Q, has been found to cause major deaths in certain parts of India (Cherian et al., 2021). A triple mutant (B.1.618) strain was recently found to cause major outbreaks and deaths in the Bengal region in India, leading to the worst COVID-19 outbreak (Huh et al., 2021).

Since the mutation rate of SARS-CoV-2 is very high, it is important to identify the major sites in its genome that show potential in mutating further and posing a risk to humankind (Chen J. et al., 2020). It is also necessary to identify the mutation types that have occurred predominately to understand the selection pressure on this novel coronavirus strain (Presti et al., 2020). In this article, we have performed mutational analysis on different proteins specific to SARS-CoV-2. We have explored the structural and functional consequences of the selected mutations on the protein structures and their interaction with respective binding partners. The association between SARS-CoV-2 virulence properties and its effect on human health has also been discussed subsequently so that different mutations that may happen in the future and their implications on humanity could be assessed.

The presence of the SARS-CoV-2 S protein assists in attaching the virus to the host cell membrane (Letko et al., 2020; Walls et al., 2020). It belongs to transmembrane (TM) glycoprotein class I and is trimeric in structure (Hoffmann et al., 2020). The activation of S protein occurs by TM protease serine 2, which is present on the host cell membrane. Post-viral entry, the release of viral RNA, translation of polyproteins and assembly of replicase-transcriptase complex for replication and transcription of viral genome occurs. This results in the synthesis of structural proteins assembled, packed and released from the host cells (Fehr and Perlman, 2015). S protein plays an important role in recognizing the ACE2 receptor, attaching virion to the host cell, and their subsequent coronavirus entry induced pathogenesis (Lan et al., 2020; Wang Q. et al., 2020). The S proteins are common to many coronaviruses and other members of the influenza family, including HIV, Ebola virus, influenza virus, paramyxovirus etc. (Hoffmann et al., 2020; Huang Y. et al., 2020).

The size and shape of the virus are maintained by the action of the most abundant structural protein in a virus referred to as M proteins (Mahtarin et al., 2020). These are membrane glycoproteins that are conserved among the β-coronaviruses (Bianchi et al., 2020). They have embedded inside the lipid bilayer and consist of an amino-terminal (NH_2_) domain at the extracellular region and a cytoplasmic domain (COOH) within the viral cell (Mousavizadeh and Ghasemi, 2020). M proteins have 222 amino acid residues in length, and they exhibit a conserved sequence suggesting a common structure of these proteins among different variants (Tang T. et al., 2020). Although higher conservation in the M protein sequence was observed among BAT-CoV, SARS-CoV and SARS-CoV-2 through multiple sequence alignment (MSA) studies, despite this, great variability was observed within the sequence of MERS-CoV suggesting their divergence from the traits shown by other members of coronaviruses (Naqvi et al., 2020).

M proteins usually interact with other (structural, non-structural, and accessory) proteins of the virus to mediate several functions. One of the main features of M protein is that it helps assemble structural proteins (S, E, and N) required for virus budding (Neuman et al., 2011; Schoeman and Fielding, 2019). These interactions between M, N, and E proteins help form virus-like particles (VLP), their intracellular trafficking, and subsequent release from the host cells (Siu et al., 2008). The stabilization of the viral RNA-N protein complex is maintained by the interaction of M with N structural proteins (Astuti, 2020). Similarly, they also interact with S protein and help in their incorporation into the virion. Their interaction is also observed during viral attachment to the host cells and in the regulation of entry processes (Naskalska et al., 2019). M proteins show self-association behavior, and their protein-protein interactions account for their ability in processing, modification and trafficking of structural proteins for assembling virus particles before release (Li et al., 2021).

Experiments involving the deletion of E protein from a highly pathogenic strain of SARS-CoV showed attenuated properties, which could be a basis of an effective vaccine against the virus (Dediego et al., 2008; Netland et al., 2010; Fett et al., 2013); however, reversions into the virulent form were reported when similar experiments were performed in cell cultures and *in-vivo* (Jimenez-Guardeño et al., 2015). Soon after, stable vaccine candidates in mice were identified by introducing deletion mutations in the C-terminal region without interrupting the PDZ binding motif (PDM) (Jimenez-Guardeño et al., 2015). Therefore, E protein serves as an excellent candidate for vaccine development in comparison to the other structural proteins (Mandala et al., 2020).

Among all structural proteins, N protein is a potent immunogen whose expression increases during infection (Shang et al., 2005; Liu et al., 2006). Most of the serological assays for the coronavirus diagnosis rely on identifying N proteins during the diagnostic procedures (Ahmed et al., 2020). More N protein-specific antibodies were detected in the serum of patients infected with SARS-CoV (Tan et al., 2004). These antibodies were more persistent and highly sensitive than other structural proteins within serum (Shi et al., 2003). Post-viral infection, these proteins bind to the viral RNA genome and play a major role in forming a ribonucleoprotein core and assist in their replication, assembly, and subsequent release from the cells to infect the new host (Ji et al., 2020). In complex with genomic RNA of the virus, N proteins provide stability and improve viral transcription and assembly (Mcbride et al., 2014). In addition to this, they also assert their role in mediating the viral life cycle (Chang et al., 2014).

To get deeper insights into the mechanism of pathogenesis, we have performed extensive sequence and structure analysis of structural and enzymatic proteins of SARS-CoV-2. The emergence of new variants and their harmful impact on human health concern healthcare experts and drug/vaccine development. In such context, our findings establish gene to disease relationships and provide the molecular basis of pathogenesis.

## Materials and Methods

### Mutational and Structural Data

The mutational data for the SARS-CoV-2 proteins, i.e., S, E, N, and M^pro^ was fetched out from the NCBI Virus database (https://www.ncbi.nlm.nih.gov/labs/virus/vssi/#/scov2_snp). The structural coordinates of all four proteins were taken from the Protein Data Bank (PDB). The sequence information was taken from the UniProt database.

### Mutational Analysis

To study the impact of the reported mutations on the S, E, N, and M^pro^ proteins structure, we have performed a structure-based analysis using various bioinformatics tools, such as DynaMut2 (Rodrigues et al., 2021), mCSM (Pires et al., 2014), CUPSAT (Ham, 2020), MAESTROweb (Laimer et al., 2016), SDM (Ju et al., 2021). MAESTROweb, mCSM, CUPSAT. SDM provides Gibbs free energy values (ΔΔ*G*); The change in free energy during the unfolding of a kinetically stable protein is described by this ΔΔ*G* value. Sometimes the mutation in proteins differentiates the free energy landscape between the mutant and the native protein. This variance in the free energy landscape is why the mutation affects the stability of a protein. DynaMut2 is based on vibrational entropy (VE); VE describes how a protein residue in an energy landscape is likely to be occupied based on average configurational entropy. A decrease in VE would increase the rigidity of a protein. If a mutation is shown destabilizing by four out of the five tools, we have considered that as a destabilizing mutation. A detailed protocol of structure-based mutational analyses can be found in our previous reports (Amir et al., 2019; Mohammad et al., 2020; Choudhury et al., 2021; Habib et al., 2021; Umair et al., 2021).

## Results

### Stabilizing and Destabilizing Mutations in SARS-CoV-2 Proteins

**Figure 1:**
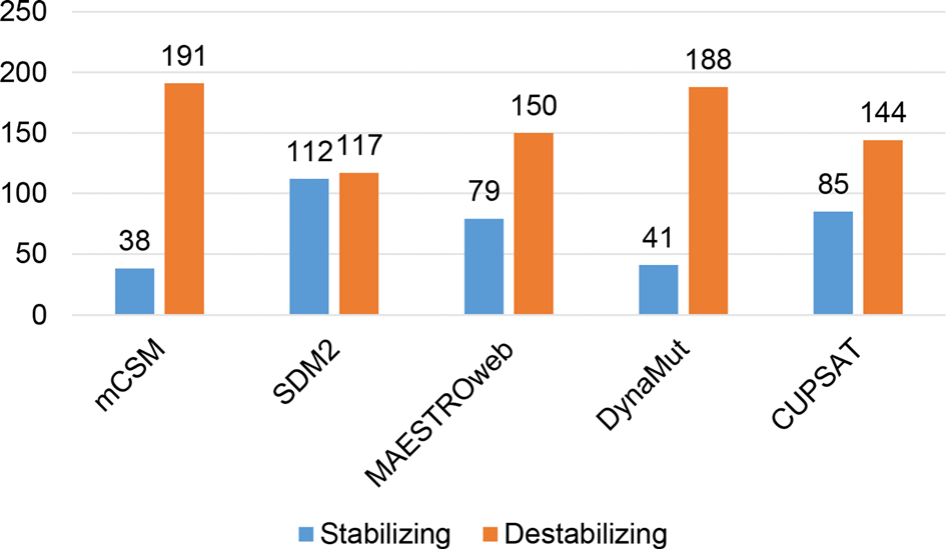
Distribution of stabilizing and destabilizing mutations in SARS-CoV-2 Spike protein predicted by structure-based tools.

**Figure 2:**
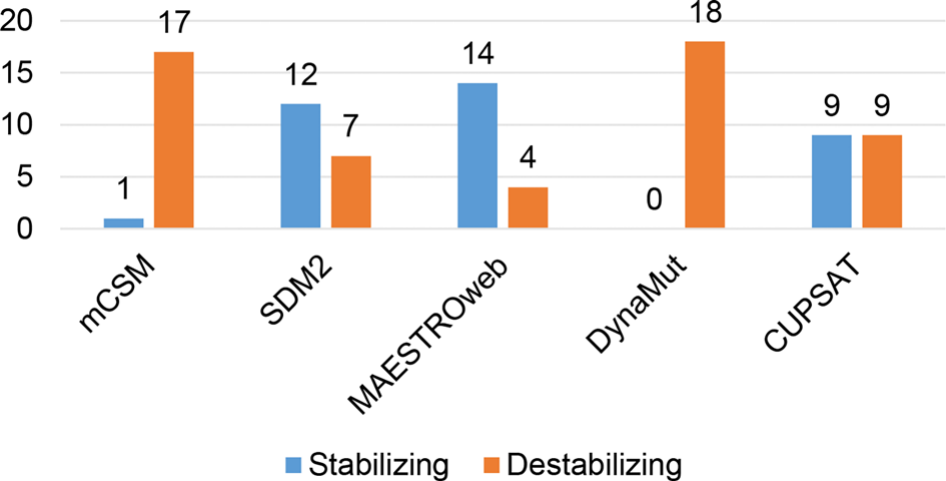
Distribution of stabilizing and destabilizing mutations in SARS-CoV-2 Envelope protein predicted by structure-based tools.

**Figure 3:**
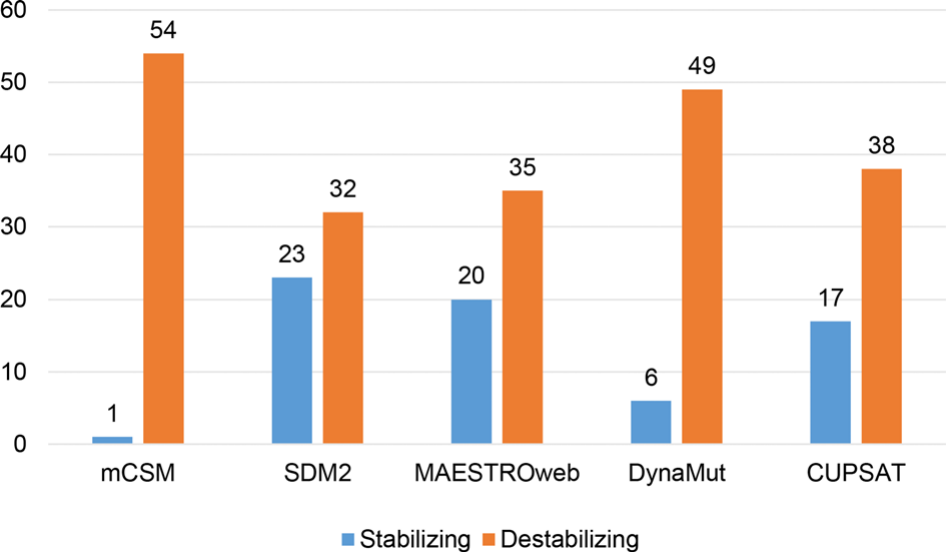
Distribution of stabilizing and destabilizing mutations in SARS-CoV-2 Main Protease predicted by structure-based tools.

**Figure 4:**
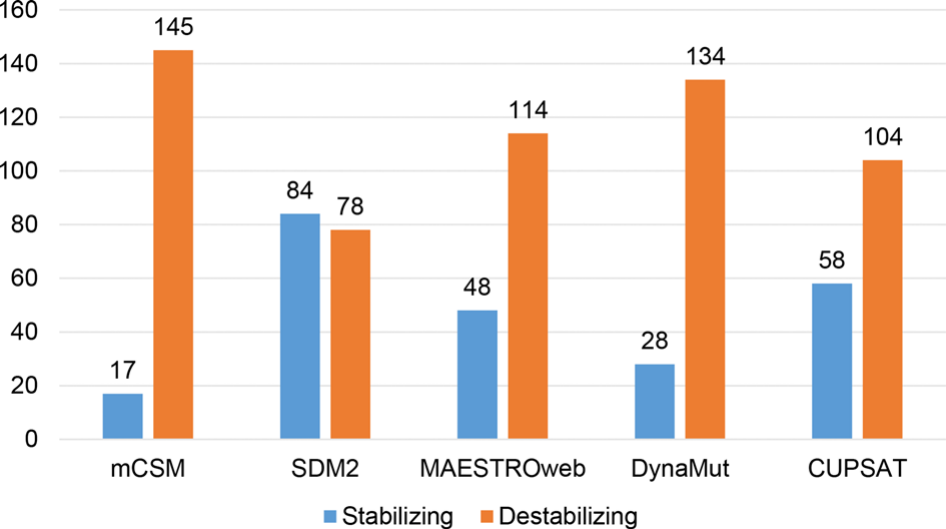
Distribution of stabilizing and destabilizing mutations in SARS-CoV-2 Nucleocapsid protein predicted by structure-based tools.

Different sets of reported mutations in the SARS-CoV-2 proteins were extracted from the NCBI Virus database. For S protein, 229 mutations were analyzed, where 123 mutations were found to be destabilizing (**Figure 1**). In contrast, 6 mutations were destabilizing out of 18 mutations in E protein (**Figure 2**). At the same time, out of 57 mutations in M^pro^, 36 mutations have a destabilizing effect (**Figure 3**). Here, 25 mutations are present in domain I and 11 mutations in domain II. While two mutations lie in the loop region, and 19 mutations are in the C-terminal domain III. The analysis of 162 N protein mutations showed 85 mutations as destabilizing (**Figure 4**). The analysis revealed that the SARS-CoV-2 structural proteins, i.e., S, E, and N, and replication machinery enzyme, i.e., M^pro^ have several mutations found in the concerning variants (**Table 1**).

**Table 1:** Selected mutations predicted to be destabilizing and stabilizing through different structure-based approaches.

Protein	Destabilizing Mutations	Stabilizing Mutations
Spike Protein	L5F, Q14H, T20I, T22N, P26S, P26L, P26T, T29I, T29P, N30K, S31T, F32L, R34P, R34S, L54F, V62L, V70I, S71F, G75V, T76I, D80Y, T95I, D111N, L141F, G142S, G142D, Y144N, Y145H, W152C, R158T, L176F, M177I, L189F, L212F, D215N, D215E, L216I, L216F, P217A, P217H, G219C, S221L, W258L, G261D, A262S, A262T, F318Y, F318I, V320F, V320I, V367F, V382L, I402V, R403K, E406D, K417N, K417E, L452R, L455F, R466G, R466I, G485R, Q498H, P499T, N501Y, N501T, L518Q, A520S, A520E, P521T, A522V, A522S, A522G, E583D, D614G, V622F, A626V, T632N, S640F, E654K, Q675H, S691F, M731I, T732A, A771S, Q779H, I788M, I794F, I794M, S810P, L822F, I834M, A845D, A845S, A846S, E868D, A879S, I882T, I882V, S884A, A899S, A930V, S939T, S940T, A942S, L945F, L1004F, Y1047N, S1055A, V1068F, A1070S, K1073N, A1078T, A1078S, V1104L, P1112L, V1122L, G1124V, N1125S, V1137I, Y1138H, P1140A, L1145F	P9L, S12F, S13I, L18F, R21I, A27V, G35C, G35R, H49Y, S50L, A67V, H69Y, D88H, S98F, D138Y, H146Y, N149Y, M153I, S155I, Q173K, E180V, G181R, V213L, R214L, R214S, D215V, D215Y, Q218L, A222V, H245Y, S247R, D253G, S254F, S255F, T307I, Q321R, Q321H, K417M, N439K, G446V, S477N, S477I, Q493K, S494P, S514C, L517P, L518I, H519Y, H519Q, H519N, K558N, Q613H, N641K, A653V, H655Y, Q677H, Q677P, P681H, P681L, R682W, R682L, A688V, S698L, A701V, S704L, T716I, T719I, G769V, V772I, T778I, Q779R, P793T, D796Y, D796H, S810L, P812S, P812L, S813T, D839E, D839Y, L841I, L841R, A845T, R847T, T859I, L894F, S929N, S929R, G932S, K933Q, D936Y, D936E, S939F, S943T, T1027I, A1078V, D1084E, D1084Y, S1097L, H1101Y, T1117I, V1133I, D1139V, L1141M, Q1142E, E1144Q
Envelope Protein	L37H, V49L, L51F, F56V, R69K, V75L	T9I, T30I, S55T, S55F, S68F, R69I, R69G, P71S, P71L, D72G, L73F, L74M
MPro	F8L, G15S, L30F, D34G, V35A, I43V, T45I, L50F, K61R, G71S, L75F, R76S, V86L, K88R, L89F, A94V, P96L, P108S, P108L, P132S, F134L, F134Y, V157L, L167F, P168S, N180K, V202F, V204A, L227F, M235L, M235I, E270A, L282F, L286F, P293S, R298K,	S10A, S10Y, L32F, V35I, S46A, L67F, K90R, A94S, T135I, N180T, A191V, T196M, A234V, S254F, A266V, S267A, R279C, A285T, L286I
Nucleocapsid	N11S, P13L, R14C, P20S, T24I, G25D, S26N, E31G, R32H, G34W, A35V, K65R, P67S, V72I, P80Q, D81Y, I94V, D103Y, A119S, G120A, I131V, L139F, D144Y, T148S, P151L, A152T, A152S, A156S, I157T, P168S, P168A, G178V, S180I, S183Y, S188P, R191L, N192D, S194L, P199S, R203K, R203S, G204R, T205I, T205N, P207L, R209S, R209K, G212S, G212V, N213K, N213D, G215D, D216E, A217T, Q229H, M234V, S235F, G238C, A251V, T265I, E290D, I292T, A308S, S327L, S327W, T334K, T334P, N345K, N345H, D348H, Q349K, A359S, T366A, E367G, P368S, A376T, E378Q, T379A, A381P, P383L, Q384H, T391I, L407F, Q409K, Q409H	Q9H, S21T, D22G, S33N, S37P, N48I, D63E, D63Y, S79G, S79T, P80T, D103E, D128E, D128Y, T135I, A152D, A173V, A182S, R185C, S187L, S188L, S190I, N192S, S193N, S193I, S194T, R195I, S197L, P199L, S202C, S202N, S202T, G204V, S206F, A208G, A208S, R209I, M210I, M210K, A211V, N213S, A220V, L230F, M234I, G243C, T247I, A267E, A267P, V270L, T271I, Q289H, H300Y, A305V, T325I, T329M, T334I, P344S, Q349H, T362I, T362K, P364L, T366I, D371Y, D377Y, T379I, P383S, Q389L, Q390P, Q390H, T393I, A398V, L400M, D401Y, D402Y, K405R, S413R, A414S

## Discussion

The SARS-CoV-2 proteins have several mutations found in the different variants emerging day by day. These mutations have various adverse impacts on the structure functions of the SARS-CoV-2 proteins making COVID-19 complex to administrate. Here we have discussed such mutations and their roles in the SARS-CoV-2 virulence.

### Spike Protein Mutations

S protein consists of 1273 amino acids and is approximately 180-200 kDa (Hoffmann et al., 2020). Several polysaccharide molecules are coated onto the surface of S protein to help escape the host’s immune response (Watanabe et al., 2020). Like other typical proteins, S protein also has an extracellular N-terminal domain (NTD), transmembrane domain and a cytoplasmic C-terminal domain (CTD) (Hwang and Yu, 2021). It has a signal peptide ranging from 1 to 13 amino acid (aa) residues followed by two different subunits, namely S1 (14-685 aa residues) and S2 (686-1273 aa residues), each one of which plays an essential role in adherence of SARS-CoV-2 to the host cells for their entry (Xia et al., 2020). In the native state, S protein is inactive. The presence of TMPRSS2 on the target cell membrane and other cellular proteases cleaves the S protein into its S1 and S2 subunits required for the fusion of viral-host membrane after the viral invasion (Bertram et al., 2013; Hoffmann et al., 2020).

S1 subunit further consists of an N-terminal region and a receptor-binding domain (RBD) (Xia et al., 2020). It initiates the viral infection cycle by binding of trimeric S protein *via* RBD of S1 subunit to ACE2 receptors on the host cell (Wrapp et al., 2020; Yan et al., 2020). This interaction induces a conformational change that directs them to form endosomes to trigger viral fusion with the host cell under the influence of low pH (Shang et al., 2020). Understanding such conformational changes provides a base for the development of drugs that disrupt the entry mechanisms (Walls et al., 2020; Shamsi et al., 2021). The atomic-level studies by cryo-electron microscopy have revealed different open and closed conformations of the RBD. These domains account for the variability of SARS-CoV-2 (Walls et al., 2020; Wrapp et al., 2020). The amino acid composition of the RBD reflects the evolutionary relatedness of SARS-CoV-2 with other members of the Coronavirus family, and it is the least conserved unit. However, the RBD of MERS-CoV was distinct, indicating the divergence from the previous strains (Andersen et al., 2020). The intermediate hosts of SARS-CoV-2 can be identified by analyzing binding affinities and the RBD domain of the S1 subunit with the ACE2 receptors (Chen Y. et al., 2020; Huang Y. et al., 2020). Mutations in the S1 region are associated with changed antigenicity, and thus, it accounts for some strains to be more infectious than others. The affinity of the receptor binding region of S1 subunit with ACE-2 receptors might change because of mutations, but their interaction is preserved among humans, cats and swine (Chan et al., 2020; Wrapp et al., 2020; Yan et al., 2020).

S2, another subunit of S protein, mediates the fusion of the virus to the host cell membrane. It consists of a fusion peptide (FP), heptapeptide repeat sequence 1 (HR1), HR2, TM domain and a cytoplasm domain at the last (Xia et al., 2020). Various large-scale structural rearrangements allow the virus to fuse with the host cell membrane (Watanabe et al., 2020). The main basis of viral-host fusion lies in the cleavage of S1 and S2 subunits, releasing the viral genome into the host cell (Tortorici et al., 2019; Rabaan et al., 2020). Multiple furin cleavage sites have been found in SARS-CoV-2, which are susceptible to attack by furin-like proteases (Millet and Whittaker, 2015; Hasan et al., 2020). These sites were absent from previously known SARS-CoV and, thus, might have increased the infectivity of SARS-CoV-2 (Coutard et al., 2020; Rabaan et al., 2020). Besides, furin-like proteases TMPRSS2 and trypsin have been found to exhibit a profound role in activating the membrane fusion domain (Heurich et al., 2014; Limburg et al., 2019; Ou et al., 2020). The fusion peptide, rich in hydrophobic residues like glycine and alanine, anchors to the host cell membrane and disrupts their lipid bilayer (Millet and Whittaker, 2018). The exposure of FP following the cleavage of S protein into two subunits and the two HR domains of the S2 subunit is important for mediating the viral fusion (Kawase et al., 2019). Certain receptor (FP) and ligand (on host cell) interactions trigger a conformational change which shortens the distance between the two membranes for fusion (Harrison, 2015). As a result, the HR1 domain becomes closer to the host cell, whereas the proximity of the HR2 domain increases with the viral membrane. The folding of HR1 and HR2 together creates a six-helical bundle structure aligned in an antiparallel form to the fusion core. In this way, viral and host cell membrane pulls each other close for bringing out the necessary fusion (Eckert and Kim, 2001). The potential mutations in the SARS-CoV-2 S protein found in different variants are discussed here:

**N501Y:** The N501Y mutation was initially found in the B.1.1.7 UK variant (Leung et al., 2021), and later it appeared in South African and Brazilian variants, which were B.1.128.1 and B.1.351, respectively (Weisblum et al., 2020). It was also detected in the Theta (P.3 or GR/1092K.V1) variant in Japan and the Philippines in February 2021. This amino acid substitution is located in the RBD of the S protein. It was found that the N501Y substitution increases the affinity of RBD to bind to the ACE2 receptor and thus enhances the transmission rate (Starr et al., 2020; Makowski et al., 2021). Our study also shows that the N501Y mutation destabilizes the SARS-CoV-2 S protein (**Table 1**).

**K417N:** This mutation is first reported in the B.1.351 variant (Beta variant or GH501Y.V2) in South Africa (Tegally et al., 2021). Later it was also detected in the P.1 variant (GR/501Y.V3, Gamma variant) in Brazil in December 2020 (Faria NR, 2021). The K417N substitution is also present in the RBD. It was found that this substitution significantly increases the binding affinity of SARS-CoV-2 RBD to the ACE2 receptor. Also, the mutation causes a huge decrease in the binding affinity STE90-C11 antibody to RBD (Fratev, 2020).

**E484K:** The E484K mutation was first reported in the South African variant B.1.351 variant (GH501Y.V2, Beta variant) (Tegally et al., 2021) and later in the P.1 variant (Gamma variant or GR/501Y.V3) (Faria NR, 2021). This mutation was also found in the Zeta (P.2), Eta (B.1.525) and Iota (B.1.526) variants (Huh et al., 2021). This mutation significantly alters the electrostatic complementarity of antibody binding to the RBD (Andreano et al., 2020). The impact of E484K mutation in the RBD was also seen in the binding of serum polyclonal neutralizing antibodies to SARS-CoV-2 (Jangra et al., 2021).

**L18F**: This mutation occurred in the South Africa B.1.351 variant (Beta variant or GH501Y.V2) [5]. Later it was also found in the P.1 variant (Gamma variant or GR/501Y.V3) and Zeta (P.2) variants in Brazil. This substitution has been found to affect the binding of neutralizing antibodies negatively [16]. Studies have shown that mutants with L18F substitution are highly sequenced variants, escaping S2L28-mediated neutralization (Mccallum et al., 2021).

**A570D:** The A570D substitution was first identified in the B.1.1.7 UK variant (Alpha or GRY GR/501Y.V1) in December 2020. Along with D614G, this mutation induces significant conformational destabilization (Socher et al., 2021).

**P681H:** This mutation was detected first in the UK B.1.1.7 UK variant. Later it was identified in P.3 (Theta or GR/1092K.V1) variant found in Japan and the Philippines in February 2021. The P681H mutation is present at a proteolytic cleavage site for furin or furin-like proteases at the junction of the fusion domain and S protein RBD (Jaimes et al., 2020). It has been shown that P681H increases cleavability at the S1/S2 junction but does not surely indicate increased membrane fusion and infectivity (Lubinski et al., 2021).

**S982A:** The S982A substitution was first identified in the B.1.1.7 UK variant (Alpha or GRY GR/501Y.V1). The S982A mutation of B.1.1.7 lineage is present on the S2 subunit of the S protein. This substitution in UK variant B.1.1.7 does not have intermolecular hydrogen bonding potential between S protein subunits (Ostrov, 2021).

**D1118H:** This mutation was detected first in the B.1.1.7 UK variant. The D1118H substitution is also present in the S2 subunit of the S protein (Chrysostomou et al., 2021). It has been suggested that this mutation can alter the stability and dynamics of trimer assembly (Zhao et al., 2021).

**A701V:** The A701V substitution was first detected in the B.1.351 variant (Beta variant or GH501Y.V2) in South Africa and later found in the Iota (B.1.526) (Annavajhala et al., 2021) variant in New York. This non-synonymous substitution is located in the cleavage site of the neighboring promoter of the S2 subunit (West et al., 2021).

**D614G:** The D614G is the most widespread mutation of the S protein; it has been found to increase the infectivity of the SARS-CoV-2 virus (Korber et al., 2020). This non-synonymous substitution was identified in the South Africa B.1.351 variant (Beta variant or GH501Y.V2), B.1.617.2 variant (Delta variant), Epsilon (B.1.427 and B.1.429) variants, Zeta (P.2), Kappa (B.1.617.1), Eta (B.1.525) and Iota (B.1.526) variants. The D614G mutation increases infectivity and enhances the replication of the SARS-CoV-2 virus in the upper respiratory tract (Plante et al., 2021). This substitution is away from the RBD. It decreases the binding affinity of S protein to the ACE2 receptor and introduces a conformational change in the S1 subunit of the S protein (Yurkovetskiy et al., 2020). D614G is present at the SD2 domain, enhancing the furin cleavage at the S1/S2 domain junction (Gobeil et al., 2021).

**R158G**: The R158G substitution was first found in the B.1.617.2 variant (Delta variant) in December 2020 in India. The mutation is absent from the RBD of the S protein (Baral et al., 2021).

**L452R**: This mutation was reported in the B.1.617.2 variant (Delta variant) and Kappa (B.1.617.1) variant in India as well as in the Epsilon (B.1.427 and B.1.429) variants in the US (Huh et al., 2021). This mutation is present in the RBD of SARS-COV-2 S protein and was found to reduce neutralizing activities in many monoclonal antibodies (Baral et al., 2021).

**T478K**: This mutation was first identified in the B.1.617.2 variant (Delta variant) in India. The mutation is also present in the RBD of the S protein (Baral et al., 2021).

**P681R**: This mutation was first detected in the B.1.617.2 variant (Delta variant) in December 2020 in India and later in the Kappa (B.1.617.1) variant. The mutation is absent from the RBD of the S protein.

### Envelope Protein Mutations

The E protein with an 8-12 kDa size is one of the smallest structural proteins in SARS-CoV-2 (Schoeman and Fielding, 2019). The sequence of E protein is highly conserved among different members of Coronaviruses. The major function of E protein involves activation of host inflammasome, viral progeny budding and release from the host cells (Nieto-Torres et al., 2015; Schoeman and Fielding, 2019). Like other structural proteins, it also possesses three distinct domains- the extracellular domain at N-terminus consisting of hydrophilic (7-12 amino acid) residues followed by a transmembrane domain of 25 hydrophobic amino acid residues and a cytosolic or C-terminus domain-containing hydrophilic amino acid sequences (Corse and Machamer, 2000; Torres et al., 2007; Surya et al., 2018). The characteristic feature of E protein is viroporin, a pentameric ion channel with no or low selectivity of ions formed from the oligomerization of the transmembrane domain (Verdiá-Báguena et al., 2012; Nieto-Torres et al., 2014). Viroporins are small M proteins that get incorporated into the host membrane and assists in the maturation and release of the viral particles (Nieva et al., 2012). Therefore, these pentameric structures with an ion-conducting pore mediate the host-pathogen interactions (Torres et al., 2006; Parthasarathy et al., 2008; Pervushin et al., 2009). Besides regulating the assembly and release of virions, they have been found to possess a significant role in the pathogenesis of the virus (Nieto-Torres et al., 2014), where pathogenesis is directly proportional to the ion channeling (IC) activity (Chellasamy et al., 2020). For example, no effect on the replication of the virus was observed after E gene knockdown, but reduced edema accumulation was witnessed. This might be because the loss of ion channeling activity of E protein resulted in the correct localization of Na^+^/K^+^ ATPase, which is probably involved in decreased edema accumulation and an increase in edema resolution. Often, the accumulation of edema is one of the reasons contributing to ARDS. Also, studies on animal models infected with IC activity lacking viruses exhibited reduced levels of IL-1β, which further reduced the production of TNF and IL-6 in the lung airways. Therefore, it was estimated that IC activity of E protein is essential in the development and progression of cytokine storm leading to permanent lung damage and ARDS in the later stages (Nieto-Torres et al., 2014).

In addition to this, the selective cation ion channel formed from viroporin is localized towards the ERGIC membrane (Wilson et al., 2004; Verdiá-Báguena et al., 2012). The C-terminal region within the E protein contains a β motif with a conserved proline amino acid residue that is important for localization into the ER-Golgi complex (Li et al., 2014; Chellasamy et al., 2020). A small part of E protein inside the host cell membrane is transferred to the virion when a virus replicates. In contrast, the larger section of this protein remains at the location of intracellular trafficking within the mammalian cells, i.e., ER-Golgi network and the ERGIC (Nieto-Torres et al., 2011; Venkatagopalan et al., 2015). Such localization of E proteins assists in viral structural assembling and budding from the host cell (Nieto-Torres et al., 2011).

In all the variants of SARS-COV-2 except the Beta (B.1.351) variant, there are no reported mutations in the E protein. The Beta variant was first discovered in the Eastern Cape province of South Africa in October 2020 (Tegally et al., 2021). India has reported more than 200 cases of the Beta variant from the time of its discovery. The potential mutation in the SARS-CoV-2 E protein found in the Beta (B.1.351) variant are discussed here:

**P71L:** It is the amino acid substitution found in the E protein of the Beta variant. Statistically, the mutation P71L was associated with disease severity and death rate. The mutation was present in deceased patients’ datasets and virus isolates of patients from high case-fatality-ratio countries (Rizwan et al., 2021).

### M^pro^ Mutations

The SARS-CoV-2 M^pro^ has several mutations reportedly found in different variants are discussed here:

**T45I:** This mutation in the domain I region of SARS-CoV-2 M^pro^ is reported in variants B.1.1.7, B.1.351, P.1, B1.617, B.1.429^+^ B.1.427 (Philot et al., 2021). It presented a polar to non-polar substitution due to which there is a reduction in its hydrogen bonding potential. SDM predicted the free energy change as stabilizing w.r.t. WT SARS-CoV-2 M^pro^. Also, Dynamut2 indicates no substantial change in the flexibility of the protein compared to the WT SARS-CoV-2 M^pro^.

**K90R:** This mutant lies in the domain-I region; it has shown relevant modifications in its potential energy concerning the WT SARS-CoV-2 M^pro^ (Philot et al., 2021). SDM has indicated this substitution as destabilizing, and the Dynamut2 score suggests the substitution will increase the flexibility of the protein. K90R mutant is reported in the variants B.1.1.7, B.1.351, P.1, B1.617, which are mainly are our variant of concern. Furthermore, a lower energy configuration and a more extensive dimeric interface have resulted from the mutant K90R (Philot et al., 2021).

**P99L:** It is reported in the B.1.1.7 variant only. The Dynamut2 score suggests the mutant will induce stability to the structure, whereas SDM shows destabilizing; a higher probability of the formation of dimeric interfaces was reported than WT SARS-CoV-2 M^pro^ (Sheik Amamuddy et al., 2020; Philot et al., 2021).

**P108S:** This mutant is reported only in the B.1.1.7 variant. The substitution showed a significant variation in potential energy (Philot et al., 2021); nevertheless, the SDM and Dynamut2 scores suggest no substantial change in the structure.

**T135I:** This substitution brought polar to non-polar replacement. It is reported in variant B.1.1.7 and P.1. SDM has predicted this as stabilizing, whereas there may be a potential increase in the protein flexibility as per Dynamut2 w.r.t. WT SARS-CoV-2 M^pro^.

**N151D:** This substitution is reported in variants B.1.1.7, B.1.351, P.1, B1.617. Compared to WT SARS-CoV-2 M^pro^, this mutant shows behavior that may induce catalysis and create distinct dimeric interfaces (Philot et al., 2021). In addition, a significant variation in potential energy was also detected (Sheik Amamuddy et al., 2020). SDM predicts destabilizing, whereas, according to Dynamut2, there will be no change in the structure.

**A234V:** The substitution A234V in the Domain III region is reported in variants B.1.1.7, B.1.351, P.1, and B.1.617. This mutant is associated with the protein’s mobility. However, it might also affect the flexibility since it lies in the highly flexible region of the protein (Sheik Amamuddy et al., 2020).

**A266V:** This mutation was found in B.1.1.7, B.1.351, P.1, and B.1.617 varients. The mutation occurred in a highly flexible region involved in protein mobility and might affect protein flexibility (Sheik Amamuddy et al., 2020). The variation might induce rigidity as per the Dynamut2 result and is also destabilizing according to SDM.

**R279C:** This mutant is also related to protein’s mobility and lies in the Domain III region. It is reported in variants B.1.1.7, B.1.351, P.1, and B.1.617. The substitution R279C increases potential energy more than WT SARS-CoV-2 M^pro^ (Philot et al., 2021). As per SDM, the mutation may destabilize the protein. However, there may be an increase in protein’s flexibility, as per Dynamut2, which might benefit the protein.

### Nucleocapsid Mutations

N proteins play a major role in packaging the viral genome into a ribonucleoprotein complex known as a capsid. This packaging ensures the proper replication and self-assembly of the virus (Chang et al., 2014). N protein contains N-terminal (NTD) and C-terminal domains (CTD) connected by a linkage region (LKR) having serine/arginine-rich (SR-rich) domain within their structural sequence (Huang et al., 2004; Hurst et al., 2009). The presence of these positive amino acid residues favors the binding of the viral genome to both the domains, i.e., NTD and CTD (Chen et al., 2007; Saikatendu et al., 2007). LKR is mostly associated with the oligomerization process (He et al., 2004a; Chang et al., 2013). This binding occurs through a long stretch of RNA binding domain in N protein consisting of approximately 140 amino acid residues (Fehr and Perlman, 2015). Structural analysis of the N protein of SARS-CoV-2 has revealed a disordered region in high content that is not in the bound state to the genomic DNA (Zeng et al., 2020). The linker region is also disordered (Chang et al., 2006; Yu et al., 2006), suggesting its ability to bind to several other partners to maintain appropriate N protein conformation (He et al., 2004a; He et al., 2004b; Luo et al., 2005). Likewise, other structural proteins, N protein of SARS-CoV-2 is also conserved among coronavirus family and shares ~ 90% sequence similarity with SARS-CoV N protein sequence (Naqvi et al., 2020). The potential mutations in the SARS-CoV-2 N protein found in different variants are discussed here:

**R203K:** Mutation R203K was observed in the B.1.1.7 variant or the alpha variant and the P.1 variant or the gamma variant, initially found in the United Kingdom and Japan/Brazil. It is found in the Ser/Arg-rick linker region (LKR), one of the protein’s most phosphorylated regions. It is one of the most found mutants in N protein. Variant R203L and G204R were aroused by homologous recombination in the SARS-CoV-2 genome (Leary et al., 2021).

**G204R:** The mutation G204R was also observed in the B.1.1.7 variant (Alpha variant) and the P.1 variant (Gamma variant), initially found in the United Kingdom and Japan/Brazil. It is also located in the LKR region. G204R, along with R203K, is one of the most found mutations in the N protein. Their presence was associated with the increase in N protein and sub-genomic RNA expression from the other ORFs (Leary et al., 2021). N protein shows the high protein intrinsic disorder, and 203/204 residue sites showed increasing entropy and their neighborhoods aligned with areas of the high disorder (Tomaszewski et al., 2020).

**T205I:** The mutation T205I was first observed in the B.1.351 variant or the beta variant of the Coronavirus, which was first observed in South Africa in October 2020. It is also found in the LKR region. T205I mutant was a common mutation at around 43% [35] since it is highly phosphorylated. Hence, the mutation disrupts the activation of N protein and thus interferes with the virus life cycle.

**S235F:** The mutation S235F was observed in the B.1.1.7 variant, I.e., the alpha variant, first observed in the United Kingdom in November 2020. It is also found in the LKR region. This mutation was seen to alter the corresponding epitopes, which can cause changes in the specificity of certain antibodies and alter the vaccine-induced protection against the disease.

Altogether, studies suggest that mutations affect pathogenesis by changing the phenotype of a protein, disrupting its stability, structure, macromolecular binding, ablation of posttranslational modification sites, etc. (Jubb et al., 2017; Joo and Liu, 2021; Padhi et al., 2021a). Some mutations increase/decrease the binding affinity of the protein towards its receptor (Lee et al., 2020). The increased binding affinity in viral proteins results in a higher infection probability (Lee et al., 2020). The N501Y mutation, located in the receptor-binding domain of the S protein, increases its binding affinity towards the ACE2 receptor (Shi et al., 2021). Various mutations in the S protein reportedly affect vaccine development, efficacy, and neutralization. The D614G mutation of S protein enhances the viral replication rate and is the most prevalent mutation, predominantly reported in B.1.617.2, B.1.427, B.1.429, P.2, B.1.617.1., B.1.525, and B.1.526 variants. It was also found that the D614G mutation may decrease binding affinity and could also change the predicted MHC binding (Akkiz, 2021; Daniloski et al., 2021). The spike protein mutations, N501Y, E484K, P681H, and K417N, found in variant B.1.1.7, B.1.351, and the B.1.128.1 could decrease the virus’s ability to attach to antibodies (Mahase, 2021). Hence, it becomes a necessity to consider the variant during the development of vaccines. However, according to some recent findings, despite the N501Y and P681H mutants in B.1.1.7, vaccine efficacy would not be affected (Seo et al., 2020; Mahase, 2021; Shen et al., 2021). The Spike mutations N501Y (B.1.1.7, B.1.351 and the B.1.128.), L18F, K417T (B.1.351), E484K (B.1.1.7, B.1.351, B.1.128.1, B.1.525 and B.1.526), and D614G (B.1.617.2, B.1.427, B.1.429, P.2, B.1.617.1., B.1.525, and B.1.526) are reported in most concerning variants and has significant inferences for evading antibody-mediated immunity (Altmann et al., 2021; Gupta, 2021; Mohammadi et al., 2021).

The T190I mutation in M^pro^ has brought a polar to non-polar substitution near the active site cavity, which might have a significant role in enzymatic activity, particularly when coupled with mutations in neighboring areas (Sheik Amamuddy et al., 2020). Another mutation adjacent to T190I is A191V; both residues belong to the most flexible regions, as substrate recognition sites often require structural flexibility to recognize the binding sites precisely (Sheik Amamuddy et al., 2020). Such mutations would alter the native conformation and activity of the SARS-CoV-2 M^pro^ and RdRp, which might affect the binding of therapeutic molecules (Padhi et al., 2021b; Padhi and Tripathi, 2021).

## Conclusion

SARS-CoV-2 is more potent than the previous strains of coronaviruses; this reflects their enormous ability to mutate into new strains. When a virus enters a host cell, it uses its machinery to replicate and synthesize viral particles. Mutations lead to the evolution of the viral genome, allowing them to better adapt and survive in the human host for their active reproduction. Such mutations are achieved by modifications in the epitopes of viral genes, making them more infective, transmissible and helps in escaping the immune responses of the host. Most of the structural proteins of SARS-CoV-2 conserved among coronavirus family and shares ~ 90% sequence similarity. However, a slight change in sequence causes a great impact on the structure and pathogenesis of SARS-CoV-2. Despite major mutations do not affect vaccine efficacy, however, sometimes become more pathogenic.

## Data Availability Statement

The original contributions presented in the study are included in the article/**Supplementary Material**. Further inquiries can be directed to the corresponding authors.

## Author Contributions

Conceptualization, TM, FA, and MIH. Methodology, AC, IH, DY, TM, and YM. Software, AC and MU. Validation, TM and MIH. Formal analysis, AC, AS, and MU. Investigation, MA, AS, and TM. Resources; data curation AC, and IH. Writing—original draft preparation, TM, PA, DY, AS, and AC. Writing—review and editing, TM, FA, and MH. Visualization, FA, AC, and IH. Supervision, MIH. Project administration, DY and MIH. Funding acquisition, FA and MIH. All authors contributed to the article and approved the submitted version.

## Funding

This work was supported by Taif University Researchers Supporting Project Number (TURSP-2020/131), Taif University, Taif, Saudi Arabia and the Indian Council of Medical Research (Grant No. ECD/Adhoc/2/2021-22).

## Conflict of Interest

The authors declare that the research was conducted in the absence of any commercial or financial relationships that could be construed as a potential conflict of interest.

## Publisher’s Note

All claims expressed in this article are solely those of the authors and do not necessarily represent those of their affiliated organizations, or those of the publisher, the editors and the reviewers. Any product that may be evaluated in this article, or claim that may be made by its manufacturer, is not guaranteed or endorsed by the publisher.
