# Supplementary material for: Genomic Variations in the Structural Proteins of SARS-CoV-2 and Their Deleterious Impact on Pathogenesis: A Comparative Genomics Approach
Source: Front Cell Infect Microbiol. 2021 Oct 13;11:765039. doi: 10.3389/fcimb.2021.765039 (PMC8548870; doi:10.3389/fcimb.2021.765039)
Supplement: Supplementary file 1 [file Table_1.docx]

**Genomic variations in the structural proteins of SARS-CoV-2 and their deleterious impact on pathogenesis: A comparative genomics approach**

**Taj Mohammad^1^, Arunabh Choudhury^2^, Insan Habib^2^, Purva Asrani^3^, Yash Mathur^2^, Mohd Umair^2^, Farah Anjum^4^, Alaa Shafie^4^, Dharmendra Kumar Yadav^5,^* and Md. Imtaiyaz Hassan^1,*^**

*^1^Centre for Interdisciplinary Research in Basic Sciences, Jamia Millia Islamia, Jamia Nagar, New Delhi 110025, INDIA.*

*^2^Department of Computer Science, Jamia Millia Islamia, New Delhi – 110025, India.*

***^3^****Department of Microbiology, University of Delhi, South Campus, New Delhi- 110021, India.*

*^4^Department of Clinical Laboratory Sciences, College of Applied Medical Sciences, Taif University, Taif, Saudi Arabia.*

*^5^College of Pharmacy, Gachon University of Medicine and Science, Hambakmoeiro, Yeonsu-gu, Incheon City, 21924, South Korea.*

**Correspondence: dharmendra30oct@gmail.com (DKY);* [*mihassan@jmi.ac.in*](mailto:mihassan@jmi.ac.in) *(M. I. H)*

**Table S1:** Structure based prediction of mutations associated with the SARS-CoV-2 spike protein.

| **S. No.** | **Mutation** | **mCSM (ΔΔ*G*)** | **Prediction** | **SDM (ΔΔ*G*)** | **Prediction** | **MaestroWeb (ΔΔ*G*)** | **DynaMut2 (ΔΔ*G*)** | **CUPSAT (ΔΔ*G* (kcal/mol))** | **Prediction** |
| --- | --- | --- | --- | --- | --- | --- | --- | --- | --- |
|  | L5F | -0.65 | Destabilizing | -0.38 | Reduced stability | -0.03201374 | -0.96 | -2.09 | Destabilizing |
|  | P9L | -0.427 | Destabilizing | -0.58 | Reduced stability | -0.061714525 | -0.84 | 1.57 | Stabilizing |
|  | S12F | -1.004 | Destabilizing | 0.64 | Increased stability | -0.105000351 | -1.51 | 1.98 | Stabilizing |
|  | S13I | -0.323 | Destabilizing | 1.23 | Increased stability | -0.048191785 | -0.8 | -1.74 | Destabilizing |
|  | Q14H | -0.999 | Destabilizing | 0.47 | Increased stability | 0.021657774 | -1.72 | -0.47 | Destabilizing |
|  | L18F | -1.011 | Destabilizing | 0.2 | Increased stability | 0.165288731 | -1.38 | 1.08 | Stabilizing |
|  | T20I | -0.285 | Destabilizing | 1.05 | Increased stability | 0.04624186 | -0.58 | -1.49 | Destabilizing |
|  | R21I | 0.395 | Stabilizing | -0.1 | Reduced stability | -0.027688596 | 0.54 | 2.82 | Stabilizing |
|  | T22N | -0.767 | Destabilizing | -0.27 | Reduced stability | 0.221738974 | -1.38 | -4.13 | Destabilizing |
|  | P26S | -1.074 | Destabilizing | -0.63 | Reduced stability | 0.127463134 | -0.76 | -1.03 | Destabilizing |
|  | P26L | -0.409 | Destabilizing | -0.58 | Reduced stability | 0.054990486 | -1.75 | -0.82 | Destabilizing |
|  | P26T | -0.967 | Destabilizing | -0.17 | Reduced stability | 0.013049784 | -1.36 | -1.47 | Destabilizing |
|  | A27V | 0.417 | Stabilizing | 1.72 | Increased stability | 0.053434974 | 1 | -3.56 | Destabilizing |
|  | T29I | -0.218 | Destabilizing | 0.08 | Increased stability | 0.134780809 | -0.51 | -1.8 | Destabilizing |
|  | T29P | -0.505 | Destabilizing | -1.8 | Reduced stability | 0.215378283 | -0.41 | 1.12 | Stabilizing |
|  | N30K | -0.131 | Destabilizing | 0.82 | Increased stability | 0.152936322 | -0.54 | -0.28 | Destabilizing |
|  | S31T | -0.492 | Destabilizing | 0.02 | Increased stability | 0.202045152 | -0.92 | -0.91 | Destabilizing |
|  | F32L | -1.214 | Destabilizing | 0.1 | Increased stability | 0.184376611 | -1.15 | -1.13 | Destabilizing |
|  | R34P | -1.426 | Destabilizing | -3.27 | Reduced stability | 0.558318475 | -2.23 | -12.29 | Destabilizing |
|  | R34S | -2.403 | Highly Destabilizing | -2.27 | Reduced stability | 0.500490465 | -0.39 | -7.7 | Destabilizing |
|  | G35C | 0.34 | Stabilizing | -1.18 | Reduced stability | 0.065981376 | 0.06 | -6.31 | Destabilizing |
|  | G35R | -0.534 | Destabilizing | -2.27 | Reduced stability | -0.001817022 | -0.85 | 2.5 | Stabilizing |
|  | H49Y | 0.781 | Stabilizing | 0.78 | Increased stability | -0.020489815 | 1.33 | -0.61 | Destabilizing |
|  | S50L | -0.044 | Destabilizing | 1.2 | Increased stability | -0.056235232 | -0.23 | -0.37 | Destabilizing |
|  | L54F | -1.28 | Destabilizing | -0.08 | Reduced stability | 0.122241345 | -1.25 | 2.84 | Stabilizing |
|  | V62L | -0.713 | Destabilizing | -1.38 | Reduced stability | 0.062939425 | -0.93 | -2.83 | Destabilizing |
|  | A67V | -0.226 | Destabilizing | 0.99 | Increased stability | 0.123301096 | -1.09 | 0.34 | Stabilizing |
|  | H69Y | 0.792 | Stabilizing | 0.78 | Increased stability | 0.058215858 | 1.22 | -2.33 | Destabilizing |
|  | V70I | -0.499 | Destabilizing | -0.27 | Reduced stability | 0.029131359 | -0.7 | 0.62 | Stabilizing |
|  | S71F | -1.036 | Destabilizing | 0.2 | Increased stability | 0.056439479 | -1.49 | -2.43 | Destabilizing |
|  | G75V | -0.379 | Destabilizing | -1.72 | Reduced stability | 0.091912381 | -1.56 | -2.71 | Destabilizing |
|  | T76I | -0.455 | Destabilizing | 1.34 | Increased stability | 0.037616156 | -0.84 | -0.83 | Destabilizing |
|  | D80Y | -0.083 | Destabilizing | -0.09 | Reduced stability | -0.014826667 | -0.04 | -0.07 | Destabilizing |
|  | D88H | 0.409 | Stabilizing | 0.21 | Increased stability | -0.03578316 | 1.21 | 2.17 | Stabilizing |
|  | T95I | -0.121 | Destabilizing | 0.07 | Increased stability | 0.08263639 | -0.37 | -1.86 | Destabilizing |
|  | S98F | -0.928 | Destabilizing | 0.64 | Increased stability | -0.040720554 | -1.49 | 4.11 | Stabilizing |
|  | D111N | -0.443 | Destabilizing | 0.3 | Increased stability | 0.153910675 | -0.9 | -2.26 | Destabilizing |
|  | D138Y | 0.206 | Stabilizing | -0.8 | Reduced stability | 0.006223179 | 0.21 | -1.05 | Destabilizing |
|  | L141F | -1.714 | Destabilizing | -0.78 | Reduced stability | 0.157685787 | -1.57 | -4.68 | Destabilizing |
|  | G142S | -1.27 | Destabilizing | -1.71 | Reduced stability | 0.176098998 | -1.14 | 0.52 | Stabilizing |
|  | G142D | -1.7 | Destabilizing | -2.54 | Reduced stability | 0.16859476 | -1.63 | 0.74 | Stabilizing |
|  | Y144N | -1.745 | Destabilizing | -1.59 | Reduced stability | 0.260862548 | -1.6 | 1.72 | Stabilizing |
|  | Y145H | -1.018 | Destabilizing | -0.2 | Reduced stability | 0.185808726 | -1.49 | 3.74 | Stabilizing |
|  | H146Y | 0.839 | Stabilizing | -0.5 | Reduced stability | 0.031997051 | 1.09 | -0.17 | Destabilizing |
|  | N149Y | -0.479 | Destabilizing | 0.82 | Increased stability | -0.147238498 | -0.57 | 1.42 | Stabilizing |
|  | W152C | -1.133 | Destabilizing | -0.3 | Reduced stability | 0.005573664 | -0.38 | -2.66 | Destabilizing |
|  | M153I | -1.104 | Destabilizing | 0.72 | Increased stability | 0.076578298 | -0.95 | 0.11 | Stabilizing |
|  | S155I | 0.138 | Stabilizing | 1.43 | Increased stability | 0.03098042 | 0.39 | -1.69 | Destabilizing |
|  | R158T | -2.057 | Highly Destabilizing | 0.21 | Increased stability | 0.079327564 | -1.59 | -0.05 | Destabilizing |
|  | Q173K | 0.059 | Stabilizing | -0.21 | Reduced stability | -0.026971164 | 0.04 | 0.98 | Stabilizing |
|  | L176F | -1.326 | Destabilizing | -0.87 | Reduced stability | 0.118444231 | -1.15 | -1.11 | Destabilizing |
|  | M177I | -0.385 | Destabilizing | 0.61 | Increased stability | 0.025471828 | -0.63 | -0.91 | Destabilizing |
|  | E180V | 0.025 | Stabilizing | 0.95 | Increased stability | 0.129746737 | 0.01 | -1.7 | Destabilizing |
|  | G181R | -0.398 | Destabilizing | -3.82 | Reduced stability | -0.169035942 | -0.59 | 0.58 | Stabilizing |
|  | L189F | -1.518 | Destabilizing | -0.58 | Reduced stability | 0.044162582 | -1.14 | -4.11 | Destabilizing |
|  | L212F | -0.868 | Destabilizing | -0.38 | Reduced stability | 0.10249209 | -0.81 | 0.32 | Stabilizing |
|  | V213L | -0.335 | Destabilizing | 0.47 | Increased stability | -0.074169886 | -0.53 | 1.08 | Stabilizing |
|  | R214L | -0.111 | Destabilizing | 0.39 | Increased stability | 0.043907414 | -1 | 0.54 | Stabilizing |
|  | R214S | -1.262 | Destabilizing | -0.18 | Reduced stability | 0.107391657 | 0.16 | 2.06 | Stabilizing |
|  | D215N | -0.509 | Destabilizing | -0.17 | Reduced stability | 0.073903738 | 0.88 | -0.55 | Destabilizing |
|  | D215V | 1.074 | Stabilizing | 0.65 | Increased stability | 0.085855771 | 0.84 | -1.83 | Destabilizing |
|  | D215E | -0.462 | Destabilizing | 0.12 | Increased stability | 0.045716646 | -1.09 | -0.43 | Destabilizing |
|  | D215Y | 1.319 | Stabilizing | 0.46 | Increased stability | 0.004585421 | -1.26 | -0.52 | Destabilizing |
|  | L216I | -0.608 | Destabilizing | -0.59 | Reduced stability | 0.202249499 | -1.34 | -1.39 | Destabilizing |
|  | L216F | -0.99 | Destabilizing | -1.2 | Reduced stability | 0.155136792 | -0.82 | 0.47 | Stabilizing |
|  | P217A | -1.092 | Destabilizing | -0.36 | Reduced stability | 0.212266484 | -1.08 | -1.59 | Destabilizing |
|  | P217H | -0.654 | Destabilizing | -0.39 | Reduced stability | 0.074934173 | -0.7 | 0.25 | Stabilizing |
|  | Q218L | 0.046 | Stabilizing | 0.56 | Increased stability | -0.076200173 | 0.23 | 0.83 | Stabilizing |
|  | G219C | -0.903 | Destabilizing | -0.24 | Reduced stability | 0.082116636 | -1.48 | 4.19 | Stabilizing |
|  | S221L | -0.216 | Destabilizing | 0.02 | Increased stability | 0.135680781 | -0.63 | -2.19 | Destabilizing |
|  | A222V | -0.208 | Destabilizing | 0.86 | Increased stability | 0.047800881 | -1.15 | 1.05 | Stabilizing |
|  | H245Y | 0.92 | Stabilizing | 0.29 | Increased stability | 0.04475425 | 1.2 | -0.78 | Destabilizing |
|  | S247R | -0.545 | Destabilizing | 1.06 | Increased stability | -0.069389533 | -0.72 | -0.58 | Destabilizing |
|  | D253G | 0.028 | Stabilizing | 0.87 | Increased stability | 0.045872259 | 0.28 | 2.28 | Stabilizing |
|  | S254F | -1.203 | Destabilizing | 0.64 | Increased stability | -0.036232807 | -1.43 | -2.61 | Destabilizing |
|  | S255F | -1.057 | Destabilizing | 0.2 | Increased stability | -0.051647798 | -1.48 | 1.18 | Stabilizing |
|  | W258L | -1.956 | Destabilizing | -1.15 | Reduced stability | 0.358447214 | -1.58 | 2.89 | Stabilizing |
|  | G261D | -0.527 | Destabilizing | -2.77 | Reduced stability | 0.1325315 | -0.61 | -1.14 | Destabilizing |
|  | A262S | -0.801 | Destabilizing | -0.14 | Reduced stability | 0.088290358 | -0.83 | -0.45 | Destabilizing |
|  | A262T | -0.823 | Destabilizing | 0.13 | Increased stability | 0.027221554 | -1.1 | -1.35 | Destabilizing |
|  | T307I | -0.252 | Destabilizing | 0.95 | Increased stability | 0.003406447 | -0.58 | 0.55 | Stabilizing |
|  | F318Y | -0.468 | Destabilizing | -0.07 | Reduced stability | 0.099844938 | -1.61 | -2.3 | Destabilizing |
|  | F318I | -1.157 | Destabilizing | -0.13 | Reduced stability | 0.337601558 | -0.25 | 0.49 | Stabilizing |
|  | V320F | -0.837 | Destabilizing | -0.38 | Reduced stability | 0.130895351 | -1.32 | -0.27 | Destabilizing |
|  | V320I | -0.372 | Destabilizing | 0.12 | Increased stability | 0.103885555 | -0.4 | -0.78 | Destabilizing |
|  | Q321R | 0.239 | Stabilizing | 0.22 | Increased stability | -0.132832895 | -0.01 | 0.83 | Stabilizing |
|  | Q321H | -0.059 | Destabilizing | 0.25 | Increased stability | -0.016005556 | 0.19 | -1.95 | Destabilizing |
|  | V367F | -0.79 | Destabilizing | -0.09 | Reduced stability | -0.191585823 | -0.5 | -0.79 | Destabilizing |
|  | V382L | -0.492 | Destabilizing | 0.23 | Increased stability | 0.134934937 | -0.64 | -0.19 | Destabilizing |
|  | I402V | -1.745 | Destabilizing | -2.15 | Reduced stability | 0.270311823 | -1.08 | -0.88 | Destabilizing |
|  | R403K | -1.167 | Destabilizing | -0.6 | Reduced stability | 0.135354789 | -2.05 | -0.77 | Destabilizing |
|  | E406D | -1.54 | Destabilizing | -1.4 | Reduced stability | 0.094492854 | -1.13 | -2.29 | Destabilizing |
|  | K417M | 0.06 | Stabilizing | -0.1 | Reduced stability | -0.165569179 | 0.06 | -1.55 | Destabilizing |
|  | K417N | -1.105 | Destabilizing | -1.34 | Reduced stability | 0.029255523 | -1.35 | -0.93 | Destabilizing |
|  | K417E | -0.483 | Destabilizing | 0.11 | Increased stability | 0.000281071 | -0.55 | -1.46 | Destabilizing |
|  | N439K | 0.186 | Stabilizing | 1.01 | Increased stability | -0.03860585 | -0.06 | -0.9 | Destabilizing |
|  | G446V | -0.411 | Destabilizing | -2.64 | Reduced stability | -0.025647397 | -1.76 | 0.57 | Stabilizing |
|  | L452R | -1.182 | Destabilizing | -0.49 | Reduced stability | 0.030778661 | -0.98 | 4.37 | Stabilizing |
|  | L455F | -1.125 | Destabilizing | -0.09 | Reduced stability | 0.17101848 | -1.33 | 0.79 | Stabilizing |
|  | R466G | -1.554 | Destabilizing | 0.21 | Increased stability | 0.307472979 | -0.46 | -0.25 | Destabilizing |
|  | R466I | -0.183 | Destabilizing | 0.29 | Increased stability | 0.084693096 | -1.71 | -1.96 | Destabilizing |
|  | S477N | -0.128 | Destabilizing | 0.22 | Increased stability | -0.154451497 | -0.14 | 0.35 | Stabilizing |
|  | S477I | -0.266 | Destabilizing | 0.23 | Increased stability | -0.07897645 | -0.65 | 1.31 | Stabilizing |
|  | G485R | -0.623 | Destabilizing | -0.15 | Reduced stability | -0.123590313 | -0.9 | -1.9 | Destabilizing |
|  | Q493K | 0.265 | Stabilizing | -0.19 | Reduced stability | -0.017951575 | 0.03 | -0.42 | Destabilizing |
|  | S494P | 0.049 | Stabilizing | -1.01 | Reduced stability | 0.066563553 | -0.06 | 1.67 | Stabilizing |
|  | Q498H | -0.851 | Destabilizing | 0.2 | Increased stability | 0.111620254 | -1.42 | -0.08 | Destabilizing |
|  | P499T | -0.646 | Destabilizing | 0.25 | Increased stability | 0.128471095 | -0.56 | -0.47 | Destabilizing |
|  | N501Y | -0.391 | Destabilizing | -0.16 | Reduced stability | 0.124524773 | -1.16 | -0.76 | Destabilizing |
|  | N501T | -0.798 | Destabilizing | 0.11 | Increased stability | 0.273852227 | -0.75 | -1.71 | Destabilizing |
|  | S514C | 0.342 | Stabilizing | 1.2 | Increased stability | 0.074854107 | 0.36 | -1.77 | Destabilizing |
|  | L517P | -0.458 | Destabilizing | -3.02 | Reduced stability | -0.054290166 | -0.12 | 5.42 | Stabilizing |
|  | L518I | -0.494 | Destabilizing | 0.72 | Increased stability | 0.081727443 | -0.64 | 0.66 | Stabilizing |
|  | L518Q | -0.424 | Destabilizing | -0.62 | Reduced stability | 0.025142776 | -0.48 | 0.02 | Stabilizing |
|  | H519Y | 1.399 | Stabilizing | -0.08 | Reduced stability | -0.081793685 | 0.11 | -0.81 | Destabilizing |
|  | H519Q | 0.128 | Stabilizing | -0.38 | Reduced stability | -0.029070102 | 1.41 | 0.22 | Stabilizing |
|  | H519N | -0.054 | Destabilizing | -0.28 | Reduced stability | -0.046901096 | 0.13 | 0.44 | Stabilizing |
|  | A520S | -0.884 | Destabilizing | -0.62 | Reduced stability | 0.066224932 | -0.87 | -0.36 | Destabilizing |
|  | A520E | -1.364 | Destabilizing | -0.74 | Reduced stability | 0.192935196 | -0.52 | -0.33 | Destabilizing |
|  | P521T | -0.456 | Destabilizing | -0.17 | Reduced stability | 0.088013682 | -0.36 | -1.36 | Destabilizing |
|  | A522V | -0.74 | Destabilizing | 0.24 | Increased stability | 0.182799804 | -1.24 | -1.56 | Destabilizing |
|  | A522S | -1.527 | Destabilizing | -1.06 | Reduced stability | 0.188059367 | -1.59 | -1.14 | Destabilizing |
|  | A522G | -1.396 | Destabilizing | -1.25 | Reduced stability | 0.306695738 | -1.53 | -0.73 | Destabilizing |
|  | K558N | -0.233 | Destabilizing | -0.45 | Reduced stability | -0.093153857 | -0.01 | 0.09 | Stabilizing |
|  | E583D | -1.29 | Destabilizing | -0.61 | Reduced stability | 0.20585909 | -1.56 | -0.28 | Destabilizing |
|  | Q613H | -1.05 | Destabilizing | 0.25 | Increased stability | 0.074369029 | -1.64 | 0.96 | Stabilizing |
|  | D614G | -0.405 | Destabilizing | 2.5 | Increased stability | 0.141723988 | -0.18 | -4.41 | Destabilizing |
|  | V622F | -0.767 | Destabilizing | -0.6 | Reduced stability | -0.06734421 | -1 | -0.14 | Destabilizing |
|  | A626V | -0.31 | Destabilizing | 1.16 | Increased stability | 0.082165148 | -1.05 | -1.34 | Destabilizing |
|  | T632N | -0.335 | Destabilizing | -0.43 | Reduced stability | 0.0797746 | -0.54 | -0.57 | Destabilizing |
|  | S640F | -0.994 | Destabilizing | 0.08 | Increased stability | 0.006494914 | -1.46 | -0.99 | Destabilizing |
|  | N641K | 0.153 | Stabilizing | 0.45 | Increased stability | 0.023131205 | 0.13 | 0.18 | Stabilizing |
|  | A653V | -0.586 | Destabilizing | 0.65 | Increased stability | 0.161726937 | -1.29 | 1.58 | Stabilizing |
|  | E654K | 0.043 | Stabilizing | -0.12 | Reduced stability | 0.043265326 | -0.13 | -1.35 | Destabilizing |
|  | H655Y | 1.205 | Stabilizing | 0.87 | Increased stability | -0.042873338 | 1.61 | -2.39 | Destabilizing |
|  | Q675H | -0.827 | Destabilizing | 0.35 | Increased stability | 0.033898912 | -1.5 | -0.65 | Destabilizing |
|  | Q677H | -0.029 | Destabilizing | 0.39 | Increased stability | -0.097410704 | 0 | -0.8 | Destabilizing |
|  | Q677P | 0.098 | Stabilizing | -0.47 | Reduced stability | 0.081319175 | 0.28 | -0.57 | Destabilizing |
|  | P681H | -0.041 | Destabilizing | -0.39 | Reduced stability | -0.108006914 | -0.65 | 1.74 | Stabilizing |
|  | P681L | -0.301 | Destabilizing | -0.58 | Reduced stability | -0.018497273 | 0.04 | -0.51 | Destabilizing |
|  | R682W | -0.562 | Destabilizing | 0.24 | Increased stability | -0.201955229 | -1.01 | 1.35 | Stabilizing |
|  | R682L | 0.304 | Stabilizing | 0.47 | Increased stability | -0.147709107 | 0.17 | 0.06 | Stabilizing |
|  | A688V | -0.456 | Destabilizing | 1.16 | Increased stability | -0.012613815 | -1.32 | -0.07 | Destabilizing |
|  | S691F | -0.971 | Destabilizing | 0.64 | Increased stability | 0.033988614 | -1.54 | -0.09 | Destabilizing |
|  | S698L | -0.355 | Destabilizing | 0.99 | Increased stability | 0.074567341 | -0.89 | 0.01 | Stabilizing |
|  | A701V | -0.387 | Destabilizing | 1.16 | Increased stability | -0.086452572 | -0.97 | -0.08 | Destabilizing |
|  | S704L | -0.274 | Destabilizing | 0.99 | Increased stability | -0.028782006 | -0.58 | -1.12 | Destabilizing |
|  | T716I | 0.01 | Stabilizing | 0.08 | Increased stability | 0.115445994 | -0.14 | 0.45 | Stabilizing |
|  | T719I | 0.185 | Stabilizing | 0.8 | Increased stability | 0.036758325 | 0.33 | -1.47 | Destabilizing |
|  | M731I | -1.303 | Destabilizing | 0.34 | Increased stability | 0.295112691 | -0.76 | -4.59 | Destabilizing |
|  | T732A | -0.704 | Destabilizing | 0.81 | Increased stability | 0.225119872 | -0.94 | -0.79 | Destabilizing |
|  | G769V | -0.491 | Destabilizing | 0.72 | Increased stability | 0.018472869 | -1.49 | 1.5 | Stabilizing |
|  | A771S | -1.449 | Destabilizing | -1.73 | Reduced stability | 0.096185035 | -1.06 | -2.19 | Destabilizing |
|  | V772I | -0.608 | Destabilizing | 0.35 | Increased stability | -0.269855986 | -0.78 | -0.69 | Destabilizing |
|  | T778I | -0.088 | Destabilizing | 0.34 | Increased stability | -0.009377107 | -0.21 | 2.51 | Stabilizing |
|  | Q779H | -0.934 | Destabilizing | -0.79 | Reduced stability | -0.085185728 | -0.82 | -0.37 | Destabilizing |
|  | Q779R | -0.334 | Destabilizing | -0.15 | Reduced stability | -0.139429258 | -1.58 | 0.13 | Stabilizing |
|  | I788M | -0.561 | Destabilizing | -0.6 | Reduced stability | 0.089640406 | -0.42 | -1.39 | Destabilizing |
|  | P793T | -0.698 | Destabilizing | 0.25 | Increased stability | 0.017782426 | -0.45 | 0.43 | Stabilizing |
|  | I794F | -0.647 | Destabilizing | -0.43 | Reduced stability | -0.109152205 | -0.41 | -1.16 | Destabilizing |
|  | I794M | -0.535 | Destabilizing | -0.6 | Reduced stability | -0.059683606 | -0.75 | -0.29 | Destabilizing |
|  | D796Y | 0.321 | Stabilizing | 0.49 | Increased stability | -0.134219758 | 0.25 | -0.17 | Destabilizing |
|  | D796H | 0.802 | Stabilizing | 0.31 | Increased stability | -0.158409187 | 1.05 | 2.2 | Stabilizing |
|  | S810L | -0.127 | Destabilizing | 0.58 | Increased stability | -0.074825356 | -0.81 | -2.42 | Destabilizing |
|  | S810P | -0.152 | Destabilizing | -0.1 | Reduced stability | 0.01763112 | -0.33 | 0.46 | Stabilizing |
|  | P812S | -0.42 | Destabilizing | 0.1 | Increased stability | -0.02001566 | -0.41 | 0.63 | Stabilizing |
|  | P812L | -0.255 | Destabilizing | 0.78 | Increased stability | -0.083213087 | -0.39 | -2.68 | Destabilizing |
|  | S813T | -0.553 | Destabilizing | 0.23 | Increased stability | 0.155943229 | -0.86 | 0.81 | Stabilizing |
|  | L822F | -1.793 | Destabilizing | -1.31 | Reduced stability | 0.169420941 | -1.65 | -1.44 | Destabilizing |
|  | I834M | -0.643 | Destabilizing | -0.6 | Reduced stability | -0.076508663 | -0.58 | -0.2 | Destabilizing |
|  | D839E | -0.53 | Destabilizing | 0.33 | Increased stability | -0.061581638 | -0.25 | 0.5 | Stabilizing |
|  | D839Y | 0.059 | Stabilizing | 0.52 | Increased stability | -0.142570959 | 0.27 | -0.55 | Destabilizing |
|  | L841I | -0.451 | Destabilizing | 0.72 | Increased stability | -0.03038961 | 0.65 | -0.54 | Destabilizing |
|  | L841R | -0.017 | Destabilizing | -0.46 | Reduced stability | -0.118358884 | -0.35 | 2.64 | Stabilizing |
|  | A845D | -0.784 | Destabilizing | -0.78 | Reduced stability | 0.127610406 | -0.14 | 0.65 | Stabilizing |
|  | A845T | -0.755 | Destabilizing | 0.13 | Increased stability | 0.028399319 | -0.27 | 0.41 | Stabilizing |
|  | A845S | -0.673 | Destabilizing | -0.14 | Reduced stability | 0.046065857 | -0.3 | 1.18 | Stabilizing |
|  | A846S | -0.68 | Destabilizing | -0.62 | Reduced stability | 0.143273417 | -0.13 | -0.26 | Destabilizing |
|  | R847T | -0.071 | Destabilizing | -0.34 | Reduced stability | 0.035644699 | 0.12 | 0.37 | Stabilizing |
|  | T859I | -0.282 | Destabilizing | 0.8 | Increased stability | 0.079685252 | -0.67 | 0.31 | Stabilizing |
|  | E868D | -0.571 | Destabilizing | -1.48 | Reduced stability | -0.178100489 | -0.37 | -0.66 | Destabilizing |
|  | A879S | -1.619 | Destabilizing | -2.18 | Reduced stability | 0.13599309 | -1.05 | -1.17 | Destabilizing |
|  | I882T | -2.104 | Highly Destabilizing | -3.12 | Reduced stability | 0.217853905 | -2.07 | -2.58 | Destabilizing |
|  | I882V | -0.684 | Destabilizing | -2.56 | Reduced stability | 0.153842964 | -0.57 | -2.85 | Destabilizing |
|  | S884A | -1.454 | Destabilizing | 1.71 | Increased stability | 0.185902566 | -1.07 | -1.54 | Destabilizing |
|  | L894F | -0.659 | Destabilizing | 0.2 | Increased stability | -0.012496569 | -0.98 | -1.56 | Destabilizing |
|  | A899S | -1.851 | Destabilizing | -1.57 | Reduced stability | 0.089675946 | -0.98 | -1.35 | Destabilizing |
|  | S929N | -0.541 | Destabilizing | 0.16 | Increased stability | -0.219484786 | -0.71 | -0.11 | Destabilizing |
|  | S929R | -0.526 | Destabilizing | 1.07 | Increased stability | -0.226225907 | -0.5 | -0.17 | Destabilizing |
|  | A930V | -0.332 | Destabilizing | -0.84 | Reduced stability | 0.082241351 | -1.22 | -0.93 | Destabilizing |
|  | G932S | -1.081 | Destabilizing | 0.18 | Increased stability | 0.080104289 | -1.24 | 2.39 | Stabilizing |
|  | K933Q | 0.007 | Stabilizing | 0.44 | Increased stability | -0.204677564 | -0.01 | -0.59 | Destabilizing |
|  | D936Y | -0.559 | Destabilizing | 0.6 | Increased stability | -0.151784424 | -0.56 | -1.55 | Destabilizing |
|  | D936E | -0.402 | Destabilizing | 1.37 | Increased stability | -0.266706016 | -1.06 | 0.41 | Stabilizing |
|  | S939F | -1.426 | Destabilizing | 0.54 | Increased stability | -0.203192458 | -1.07 | -0.83 | Destabilizing |
|  | S939T | -0.677 | Destabilizing | -0.21 | Reduced stability | 0.007927042 | -1.09 | -0.78 | Destabilizing |
|  | S940T | -0.41 | Destabilizing | -0.69 | Reduced stability | 0.097436883 | -0.91 | 0.7 | Stabilizing |
|  | A942S | -0.717 | Destabilizing | -0.39 | Reduced stability | 0.091190847 | -0.25 | 0.43 | Stabilizing |
|  | S943T | -0.253 | Destabilizing | -0.14 | Reduced stability | -0.227737605 | -0.43 | 0.54 | Stabilizing |
|  | L945F | -1.371 | Destabilizing | -0.52 | Reduced stability | 0.16660676 | -1.29 | -1.04 | Destabilizing |
|  | L1004F | -1.598 | Destabilizing | -1.31 | Reduced stability | 0.180576284 | -1.55 | 1.65 | Stabilizing |
|  | T1027I | -0.412 | Destabilizing | 1.51 | Increased stability | -0.051644314 | -0.89 | 1.23 | Stabilizing |
|  | Y1047N | -1.889 | Destabilizing | -1.59 | Reduced stability | 0.108801601 | -2.22 | -1.2 | Destabilizing |
|  | S1055A | -0.938 | Destabilizing | 0.13 | Increased stability | 0.091524405 | -1.59 | -1.11 | Destabilizing |
|  | V1068F | -0.93 | Destabilizing | -1.02 | Reduced stability | 0.062218519 | -1.5 | 1.82 | Stabilizing |
|  | A1070S | -1.176 | Destabilizing | -0.24 | Reduced stability | 0.047044562 | -0.58 | -2.31 | Destabilizing |
|  | K1073N | -1.049 | Destabilizing | -0.81 | Reduced stability | 0.000902025 | -1.79 | -2.24 | Destabilizing |
|  | A1078V | 0.499 | Stabilizing | 0.99 | Increased stability | 0.161533155 | -0.97 | -0.8 | Destabilizing |
|  | A1078T | -0.875 | Destabilizing | -1.33 | Reduced stability | 0.174114043 | -1.08 | -3.85 | Destabilizing |
|  | A1078S | -1.227 | Destabilizing | -1.8 | Reduced stability | 0.287510877 | 0.83 | -4.79 | Destabilizing |
|  | D1084E | -0.01 | Destabilizing | 0.06 | Increased stability | -0.032017684 | -0.19 | -0.84 | Destabilizing |
|  | D1084Y | -0.051 | Destabilizing | -0.14 | Reduced stability | -0.148517221 | 0.02 | 3.01 | Stabilizing |
|  | S1097L | -0.18 | Destabilizing | 0.22 | Increased stability | 0.142624678 | -0.6 | 0.76 | Stabilizing |
|  | H1101Y | 1.29 | Stabilizing | 0.29 | Increased stability | -0.085048358 | 1.53 | -0.46 | Destabilizing |
|  | V1104L | -0.49 | Destabilizing | -1.41 | Reduced stability | 0.146071448 | -0.69 | -3.09 | Destabilizing |
|  | P1112L | -0.337 | Destabilizing | -0.58 | Reduced stability | 0.015731887 | -0.68 | -1.72 | Destabilizing |
|  | T1117I | -0.187 | Destabilizing | 0.07 | Increased stability | -0.172221002 | -0.5 | 1.21 | Stabilizing |
|  | V1122L | -0.456 | Destabilizing | -0.77 | Reduced stability | 0.02607636 | -0.69 | -0.63 | Destabilizing |
|  | G1124V | -0.201 | Destabilizing | -1.72 | Reduced stability | -0.021947396 | -0.87 | -4.28 | Destabilizing |
|  | N1125S | -0.019 | Destabilizing | -0.16 | Reduced stability | 0.16847293 | 0.33 | -0.78 | Destabilizing |
|  | V1133I | -0.462 | Destabilizing | 0.08 | Increased stability | 0.138704443 | -0.42 | 0.15 | Stabilizing |
|  | V1137I | -0.846 | Destabilizing | -0.34 | Reduced stability | 0.112255674 | -0.91 | 1.66 | Stabilizing |
|  | Y1138H | -0.715 | Destabilizing | -0.22 | Reduced stability | 0.083403244 | -0.3 | -0.9 | Destabilizing |
|  | D1139V | 0.224 | Stabilizing | 0.77 | Increased stability | 0.156398736 | 0.03 | 0.8 | Stabilizing |
|  | P1140A | -1.61 | Destabilizing | 0.67 | Increased stability | 0.190663044 | -1.76 | -0.47 | Destabilizing |
|  | L1141M | -0.502 | Destabilizing | -0.31 | Reduced stability | -0.029558391 | -0.33 | 1.1 | Stabilizing |
|  | Q1142E | -0.156 | Destabilizing | 0.4 | Increased stability | -0.18335763 | -0.07 | -0.16 | Destabilizing |
|  | E1144Q | -0.474 | Destabilizing | -0.43 | Reduced stability | -0.236245215 | -0.77 | 0.03 | Stabilizing |
|  | L1145F | -0.805 | Destabilizing | -0.63 | Reduced stability | -0.330921076 | -0.69 | -0.14 | Destabilizing |

**Table S2:** Structure based prediction of mutations associated with the SARS-CoV-2 envelope protein.

| **S. No.** | **Substitution** | **mCSM (ΔΔ*G*)** | **mCSM Result** | **SDM (ΔΔ*G*)** | **SDM Result** | **MaestroWeb (ΔΔ*G*)** | **MaestroWeb Result** | **CUPSAT (ΔΔ*G*)** | **CUPSAT Result** | **DynaMut2 (ΔΔ*G*)** | **DynaMut2 Result** |
| --- | --- | --- | --- | --- | --- | --- | --- | --- | --- | --- | --- |
|  | L51F | -1.022 | Destabilizing | -0.07 | Reduced stability | -0.451228757 | Stabilizing | -1.18 | Destabilizing | -0.7 | Destabilizing |
|  | R69I | -0.161 | Destabilizing | 0.87 | Increased stability | -0.401574065 | Stabilizing | -1.02 | Destabilizing | -0.35 | Destabilizing |
|  | S55T | -0.107 | Destabilizing | -0.56 | Reduced stability | -0.09742893 | Stabilizing | 0.02 | Stabilizing | -0.06 | Destabilizing |
|  | F56V | -0.954 | Destabilizing | 0.1 | Increased stability | 0.288727862 | Destabilizing | -0.19 | Destabilizing | -0.45 | Destabilizing |
|  | R69K | -0.889 | Destabilizing | -0.05 | Reduced stability | -0.381622694 | Stabilizing | -1.02 | Destabilizing | -1.18 | Destabilizing |
|  | P71S | -0.684 | Destabilizing | 0.94 | Increased stability | -0.459593142 | Stabilizing | 0.29 | Stabilizing | -0.28 | Destabilizing |
|  | L74M | -0.734 | Destabilizing | -0.32 | Reduced stability | -0.741261827 | Stabilizing | 1.27 | Stabilizing | -0.48 | Destabilizing |
|  | L37H | -0.095 | Destabilizing | -0.98 | Reduced stability | 1.260743086 | Destabilizing | -1.38 | Destabilizing | -0.02 | Destabilizing |
|  | V75L | -0.416 | Destabilizing | -0.59 | Reduced stability | -0.600819892 | Stabilizing | -0.3 | Destabilizing | -0.27 | Destabilizing |
|  | S68F | -1.115 | Destabilizing | 0.64 | Increased stability | -0.297441039 | Stabilizing | -0.75 | Destabilizing | -0.96 | Destabilizing |
|  | L73F | -0.732 | Destabilizing | 0.18 | Increased stability | -0.179234196 | Stabilizing | -0.77 | Destabilizing | -0.66 | Destabilizing |
|  | R69G | -1.272 | Destabilizing | 2.38 | Increased stability | 0.571466661 | Destabilizing | 0.68 | Stabilizing | -1.38 | Destabilizing |
|  | T30I | 0.014 | Stabilizing | 1.51 | Increased stability | -0.632644426 | Stabilizing | 0 | No change | -0.03 | Destabilizing |
|  | D72G | -0.802 | Destabilizing | 2.06 | Increased stability | 0.072864886 | Destabilizing | 0.23 | Stabilizing | -0.52 | Destabilizing |
|  | V49L | -0.748 | Destabilizing | -0.21 | Reduced stability | -0.808032699 | Stabilizing | -0.08 | Destabilizing | -0.82 | Destabilizing |
|  | S55F | -0.8 | Destabilizing | 0.43 | Increased stability | -0.146554302 | Stabilizing | 0.75 | Stabilizing | -0.79 | Destabilizing |
|  | T9I | -0.171 | Destabilizing | 1.31 | Increased stability | -0.659224862 | Stabilizing | 0.45 | Stabilizing | -0.34 | Destabilizing |
|  | P71L | -0.36 | Destabilizing | 2.12 | Increased stability | -0.096425656 | Stabilizing | 0.56 | Stabilizing | -0.38 | Destabilizing |

**Table S3:** Structure based prediction of mutations associated with the SARS-CoV-2 main protease.

| **S. No.** | **Variations** | **McSM** | **Cupsat** | **MAESTRO** | **DYNAMUT-2** | **SDM-2** |
| --- | --- | --- | --- | --- | --- | --- |
|  |  | Pred_DDG | Predicted DDG (kcal/mol) | Pred_DDG | Pred_Score | DDG |
|  | F8L | -0.977 | -1.41 | 0.88944327 | -0.57 | -1.46 |
|  | S10A | -0.481 | -1.2 | -0.588822457 | -0.65 | 0.11 |
|  | S10Y | -0.468 | -0.54 | -0.981000572 | 0.02 | -0.59 |
|  | G15S | -1.041 | 0.19 | 0.427159297 | -0.61 | -0.79 |
|  | L30F | -1.639 | -3 | -0.238277161 | -1.77 | -0.66 |
|  | L32F | -1.398 | -0.5 | -0.242661379 | -0.77 | -0.66 |
|  | D34G | -0.936 | -0.88 | 0.438805331 | -0.34 | -0.58 |
|  | V35I | -0.727 | 1.82 | -0.904749769 | -0.34 | -0.28 |
|  | V35A | -2.048 | -2.38 | 0.639562026 | -1.89 | -1.74 |
|  | I43V | -1.583 | -1.68 | 1.101752763 | -1.09 | -2.85 |
|  | T45I | -0.286 | -1.36 | 1.082189581 | -0.21 | 0.36 |
|  | S46A | -0.247 | 2.45 | -0.120052934 | -0.06 | 0.86 |
|  | L50F | -1.193 | 0.51 | 0.494336413 | -0.85 | -0.57 |
|  | K61R | -1.169 | -0.84 | 0.282618751 | -0.06 | 0.38 |
|  | L67F | -0.975 | -1.33 | -0.114072103 | -0.35 | -0.1 |
|  | G71S | -0.541 | 2.04 | 0.056342753 | -0.1 | -2.58 |
|  | L75F | -1.465 | -0.59 | 0.499761462 | -0.64 | -0.75 |
|  | R76S | -0.175 | 0.51 | 1.780550744 | -0.27 | -0.87 |
|  | V86L | -1.057 | -1.21 | -0.782421206 | -0.81 | -1.5 |
|  | K88R | -0.871 | -1.93 | -0.762255413 | -0.09 | -0.21 |
|  | L89F | -1.851 | -1.13 | -0.533155808 | -1.67 | -0.66 |
|  | K90R | -0.887 | 1.09 | -0.174122424 | 0.2 | -0.21 |
|  | A94S | -1.14 | 0.79 | -0.000349018 | -0.44 | -1.14 |
|  | A94V | -0.647 | -0.55 | -0.053818438 | -1.08 | -0.04 |
|  | P96L | -0.369 | -1.85 | 0.60278841 | -0.41 | 0.84 |
|  | P108S | -1.48 | -0.71 | 0.759948425 | -0.28 | -0.55 |
|  | P108L | -0.526 | -0.16 | -0.161823101 | -1.05 | -0.07 |
|  | P132S | -2.109 | -1.23 | 0.394653643 | -1.63 | -0.77 |
|  | F134L | -0.965 | -0.84 | 0.344368024 | -0.42 | 0.26 |
|  | F134Y | -0.399 | -1.79 | 0.216195462 | -0.49 | -0.21 |
|  | T135I | -0.09 | -1.67 | -0.893718921 | 0.15 | 0.6 |
|  | V157L | -0.382 | -2.09 | -0.040071909 | -0.35 | -1.5 |
|  | L167F | -1.487 | -0.07 | -0.419646252 | -1.04 | -1.31 |
|  | P168S | -0.474 | -3.45 | 0.956144869 | -0.09 | -0.26 |
|  | N180T | -0.721 | -0.01 | -0.277624953 | -0.15 | 0.15 |
|  | N180K | -0.252 | -0.79 | 0.217463269 | -0.53 | -0.19 |
|  | A191V | -0.327 | 2.27 | 0.481107687 | -0.99 | -0.04 |
|  | T196M | -0.1 | 0.02 | 0.810602828 | -0.01 | 0.43 |
|  | V202F | -1.398 | -1.35 | 0.64292521 | -1.68 | -1.08 |
|  | V204A | -2.276 | -4.21 | 1.517550356 | -2.12 | -0.69 |
|  | L227F | -1.299 | -1.2 | 0.539854755 | -0.53 | -0.69 |
|  | A234V | 0.204 | 0.98 | 0.829841993 | 0.25 | -1.03 |
|  | M235L | -0.377 | -0.52 | 0.009595406 | -0.29 | 0.35 |
|  | M235I | -0.377 | -1.55 | 0.128977232 | -0.27 | 0.08 |
|  | S254F | -1.032 | 1.49 | -0.890798974 | -0.75 | 1.02 |
|  | A266V | -0.5 | 0.01 | 1.398220957 | -1.13 | -1.03 |
|  | S267A | -0.893 | 0.97 | 0.280260813 | -0.46 | 2.11 |
|  | E270A | -0.993 | -0.96 | 0.851561374 | -0.76 | 0.73 |
|  | R279C | -0.681 | 0.25 | 2.179296059 | 0.11 | -0.24 |
|  | L282F | -1.132 | -0.72 | 0.362002644 | -0.77 | -0.76 |
|  | A285T | -0.721 | 0.6 | -0.090426466 | 0.2 | -0.31 |
|  | L286I | -0.367 | 0.5 | 1.031076199 | -0.65 | 0.45 |
|  | L286F | -0.591 | -1.71 | 0.625356352 | -0.31 | -0.15 |
|  | P293S | -2.244 | -1.79 | 0.943326672 | -1.76 | 0.03 |
|  | R298K | -1.185 | -0.38 | 0.578711763 | -1.69 | -1.16 |

**Table S4:** Structure based prediction of mutations associated with the SARS-CoV-2 nucleocapsid protein**.**

| **S. No.** | **Mutations** | **mCSM** | **Maestro ΔΔ*G* pred.** | **CupSat** | **Overall stability** | **SDM2** |
| --- | --- | --- | --- | --- | --- | --- |
|  | R203K | -1.199 | 0.895 | Destablising | Unfavourable | -0.41 |
|  | G204R | -0.111 | 0.39 | Destablising | Unfavourable | -0.17 |
|  | S194L | -0.401 | 0.156 | Destablising | Unfavourable | 1.25 |
|  | P199L | -0.662 | -0.048 | Stablising | Unfavourable | 1.8 |
|  | P67S | -1.505 | 0.271 | Stablising | Unfavourable | -0.63 |
|  | P13L | -0.331 | 0.686 | Stablising | Unfavourable | -0.04 |
|  | T205I | -0.333 | 0.227 | Destablising | Unfavourable | 1.26 |
|  | S197L | -0.469 | -0.057 | Destablising | Unfavourable | 1.44 |
|  | L230F | -1.429 | -0.345 | Stablising | Unfavourable | -0.88 |
|  | S183Y | -0.679 | 0.403 | Destablising | Unfavourable | 0.94 |
|  | I292T | -2.555 | 0.063 | Destablising | Unfavourable | -3.46 |
|  | R209I | 0.098 | 1.477 | Destablising | Unfavourable | 0.58 |
|  | A208G | -0.388 | -0.052 | Stablising | Favourable | 1.84 |
|  | M234I | 0.062 | 0.951 | Destablising | Favourable | 0.31 |
|  | G212S | -0.608 | 0.146 | Destablising | Unfavourable | -3.15 |
|  | G25D | -0.462 | 0.152 | Stablising | Unfavourable | -2.33 |
|  | T391I | -0.034 | 0.021 | Destablising | Unfavourable | 1.52 |
|  | G34W | -1.194 | 0.158 | Destablising | Favourable | -0.2 |
|  | D22G | -0.029 | 0.883 | Destablising | Unfavourable | 0.8 |
|  | S26N | -0.022 | 0.173 | Destablising | Unfavourable | 0.39 |
|  | Q349K | -0.387 | 0.734 | Destablising | Favourable | -0.49 |
|  | A376T | -0.897 | 0.652 | Destablising | Favourable | -1.81 |
|  | S193N | -0.049 | -0.002 | Destablising | Unfavourable | 0.83 |
|  | A267E | -0.823 | -0.703 | Stablising | Favourable | 0.14 |
|  | S37P | -0.485 | -0.067 | Stablising | Favourable | -0.25 |
|  | A152D | -3.331 | 1.083 | Stablising | Favourable | -1.92 |
|  | T334K | -0.826 | 1.118 | Destablising | Unfavourable | -1.35 |
|  | D128E | -0.305 | 0.543 | Stablising | Favourable | 0.2 |
|  | T205N | -0.779 | 0.232 | Destablising | Unfavourable | -0.2 |
|  | A267P | -0.399 | -0.273 | Stablising | Unfavourable | -0.69 |
|  | N345K | -0.32 | 1.054 | Stablising | Favourable | -0.24 |
|  | D377Y | 0.041 | 0.45 | Destablising | Unfavourable | 0.26 |
|  | E31G | -0.37 | 1.816 | Destablising | Favourable | 2.43 |
|  | A152T | -1.649 | 0.157 | Destablising | Unfavourable | -1.95 |
|  | S190I | -0.496 | -0.171 | Stablising | Unfavourable | 1.59 |
|  | M210I | -0.064 | 1.537 | Stablising | Unfavourable | 0.55 |
|  | D63E | 0.31 | 0.304 | Destablising | Unfavourable | 0.08 |
|  | A152S | -1.78 | 0.191 | Destablising | Favourable | -2.4 |
|  | A217T | -0.573 | -0.518 | Destablising | Unfavourable | -0.31 |
|  | Q349H | -1.085 | 0.23 | Destablising | Unfavourable | 1.12 |
|  | S21T | -0.322 | 0.787 | Stablising | Favourable | 0.76 |
|  | K65R | -0.37 | 0.39 | Destablising | Unfavourable | -0.11 |
|  | I94V | -1.393 | -0.195 | Destablising | Favourable | -2.56 |
|  | S33N | -0.222 | 0.293 | Stablising | Unfavourable | 0.78 |
|  | Q409K | -0.011 | 0.517 | Destablising | Favourable | -0.21 |
|  | A381P | -0.3 | 0.308 | Destablising | Favourable | -0.22 |
|  | I157T | -2.255 | 0.651 | Destablising | Favourable | -2.45 |
|  | D216E | -0.245 | -0.321 | Destablising | Favourable | -0.23 |
|  | T334P | -0.675 | 0.16 | Destablising | Unfavourable | -1.99 |
|  | N345H | -0.877 | 0.467 | Destablising | Favourable | 0.82 |
|  | G120A | -0.405 | 0.021 | Stablising | Favourable | -0.12 |
|  | N192S | -0.004 | 0.94 | Stablising | Favourable | -0.8 |
|  | E290D | -1.521 | -0.302 | Destablising | Unfavourable | -1 |
|  | S79G | 0.217 | 0.085 | Stablising | Favourable | 2.53 |
|  | D103E | -0.762 | 0.533 | Stablising | Unfavourable | 0.25 |
|  | I131V | -0.936 | 0.458 | Destablising | Favourable | 0.29 |
|  | N192D | -0.23 | 0.51 | Stablising | Favourable | -0.02 |
|  | Q229H | -0.841 | 0.054 | Destablising | Unfavourable | 0.56 |
|  | M234V | -0.011 | 1.079 | Destablising | Unfavourable | 0.25 |
|  | N213K | -0.095 | 0.975 | Destablising | Unfavourable | 0.49 |
|  | L400M | -0.675 | -0.386 | Stablising | Favourable | -0.8 |
|  | T379A | -0.408 | 0.268 | Destablising | Unfavourable | 0.75 |
|  | N213D | -0.434 | 0.1 | Destablising | Unfavourable | -0.05 |
|  | N213S | -0.418 | 0.49 | Stablising | Favourable | -0.51 |
|  | Q390P | 0.086 | 1.803 | Destablising | Unfavourable | -0.95 |
|  | Q390H | 0.046 | 1.229 | Destablising | Favourable | 1.14 |
|  | Q409H | -0.748 | 0.103 | Destablising | Unfavourable | 1.15 |
|  | K405R | -0.751 | -0.067 | Stablising | Unfavourable | 0.14 |
|  | L407F | -1.153 | -0.458 | Destablising | Unfavourable | -0.57 |
|  | A35V | -0.321 | 0.145 | Stablising | Unfavourable | -0.21 |
|  | P151L | -0.327 | 0.424 | Destablising | Unfavourable | 1.85 |
|  | A182S | -0.324 | -0.482 | Destablising | Favourable | -0.84 |
|  | R209S | -0.551 | 0.452 | Destablising | Favourable | -0.59 |
|  | P383L | -0.286 | 0.319 | Destablising | Favourable | 1.29 |
|  | M210K | 0.428 | 1.169 | Stablising | Favourable | -0.08 |
|  | R209K | -0.529 | 0.906 | Destablising | Favourable | -0.1 |
|  | S202C | -0.634 | -0.229 | Destablising | Favourable | 1.16 |
|  | G212V | -0.528 | -0.081 | Destablising | Unfavourable | -1.45 |
|  | N11S | -0.205 | 0.624 | Destablising | Unfavourable | -1.21 |
|  | D103Y | -0.056 | 0.252 | Destablising | Unfavourable | -0.01 |
|  | S235F | -1.03 | 0.406 | Destablising | Unfavourable | 1.16 |
|  | Q289H | -0.763 | -0.026 | Destablising | Unfavourable | 0.84 |
|  | G238C | -0.908 | 0.364 | Destablising | Favourable | -0.42 |
|  | A119S | -0.962 | 0.72 | Destablising | Unfavourable | -1.82 |
|  | A156S | -0.985 | 0.198 | Destablising | Unfavourable | -2 |
|  | S193I | -0.457 | -0.175 | Destablising | Favourable | 2.18 |
|  | A211V | -0.459 | 0.138 | Stablising | Unfavourable | 0.39 |
|  | S413R | -0.126 | -0.148 | Stablising | Favourable | 1.4 |
|  | R32H | -1.764 | 1.315 | Destablising | Favourable | 0.3 |
|  | T334I | -0.569 | -0.321 | Destablising | Favourable | 1.1 |
|  | V72I | -0.368 | 0.062 | Destablising | Unfavourable | -0.18 |
|  | D144Y | -0.258 | 0.265 | Destablising | Unfavourable | 0.17 |
|  | S194T | -0.222 | 0.209 | Stablising | Unfavourable | 0.68 |
|  | P199S | -0.969 | 0.269 | Destablising | Unfavourable | -0.55 |
|  | S180I | -0.156 | 0.69 | Destablising | Favourable | 1.03 |
|  | D371Y | 0.09 | 0.429 | Destablising | Favourable | -0.43 |
|  | D401Y | -1.302 | -0.386 | Stablising | Unfavourable | -1.04 |
|  | T135I | -0.312 | -0.067 | Destablising | Unfavourable | 0.48 |
|  | P168S | -1.878 | 0.193 | Destablising | Unfavourable | -1.17 |
|  | S202N | -0.499 | -0.091 | Stablising | Favourable | 0.28 |
|  | P168A | -1.601 | 0.269 | Destablising | Unfavourable | -0.49 |
|  | H300Y | 0.464 | 0.153 | Stablising | Favourable | -0.38 |
|  | Q9H | -0.468 | 0.871 | Stablising | Favourable | 0.83 |
|  | R14C | -0.264 | 2.119 | Destablising | Favourable | -0.15 |
|  | T24I | -0.369 | 0.609 | Destablising | Unfavourable | 1.07 |
|  | N48I | 1.054 | 0.194 | Stablising | Unfavourable | 1.24 |
|  | D128Y | 0.348 | 0.524 | Destablising | Unfavourable | 0.51 |
|  | R203S | -1.352 | 1.312 | Stablising | Unfavourable | -1.56 |
|  | P207L | -0.2 | 0.7 | Destablising | Unfavourable | 1.37 |
|  | A220V | -0.297 | -0.29 | Stablising | Favourable | -1.03 |
|  | A251V | -0.229 | 0.306 | Destablising | Unfavourable | -0.04 |
|  | S327L | -0.429 | 1.119 | Destablising | Unfavourable | 2.16 |
|  | T393I | -0.347 | 0.844 | Stablising | Favourable | 1.06 |
|  | L139F | -0.764 | 0.342 | Destablising | Unfavourable | -0.19 |
|  | T379I | 0.179 | -0.431 | Destablising | Favourable | 1.82 |
|  | E367G | -1.036 | 0.418 | Destablising | Unfavourable | -0.25 |
|  | V270L | -0.974 | -0.303 | Destablising | Favourable | 0.21 |
|  | S202T | -0.321 | 0.142 | Stablising | Unfavourable | 0.45 |
|  | T247I | -0.154 | 0.799 | No Change | Favourable | 0.46 |
|  | T362I | -0.171 | 0.052 | Stablising | Unfavourable | 2.74 |
|  | Q384H | -0.074 | 0.313 | Destablising | Unfavourable | 0.7 |
|  | S206F | -1.126 | -0.075 | Destablising | Unfavourable | 0.87 |
|  | T271I | -0.205 | -0.396 | Stablising | Favourable | 1.28 |
|  | D348H | -0.963 | 0.295 | Destablising | Unfavourable | 0.67 |
|  | P364L | -0.323 | 0.994 | Stablising | Favourable | 2.85 |
|  | P383S | -0.506 | -0.412 | Destablising | Unfavourable | 0.04 |
|  | T366A | -0.477 | 0.718 | Destablising | Unfavourable | 1.15 |
|  | Q389L | 0.211 | 1.183 | Destablising | Favourable | 1.4 |
|  | D402Y | -1.401 | -0.17 | Destablising | Favourable | 0.56 |
|  | R185C | -0.396 | 0.89 | Stablising | Unfavourable | -0.45 |
|  | S187L | -0.463 | -1.121 | Stablising | Favourable | 1.34 |
|  | P344S | -0.695 | -0.326 | Stablising | Favourable | -0.26 |
|  | T362K | -0.388 | -0.189 | Stablising | Favourable | -0.2 |
|  | P80T | -0.751 | 0.311 | Stablising | Unfavourable | 0.16 |
|  | R191L | -0.982 | 0.29 | Destablising | Unfavourable | 0.71 |
|  | D63Y | 1.457 | 0.097 | Destablising | Unfavourable | 0.02 |
|  | T148S | -1.383 | 0.369 | Destablising | Unfavourable | -0.7 |
|  | S188L | -0.204 | 0.313 | Stablising | Unfavourable | 1.39 |
|  | G215D | -1.3 | -0.7 | Destablising | Unfavourable | -2.8 |
|  | T265I | -0.177 | 0.205 | Destablising | Unfavourable | 1.47 |
|  | A308S | -0.786 | -0.368 | Destablising | Favourable | -1.26 |
|  | E378Q | -0.393 | 0.476 | Destablising | Unfavourable | -0.89 |
|  | P80Q | -0.781 | 0.024 | Stablising | Favourable | -0.23 |
|  | A173V | 0.457 | -0.159 | Destablising | Unfavourable | -0.04 |
|  | R195I | -0.63 | 1.412 | Stablising | Favourable | 1.21 |
|  | G204V | -0.425 | -0.097 | Stablising | Unfavourable | 0.16 |
|  | G243C | -0.847 | -0.171 | Destablising | Favourable | 0.18 |
|  | T325I | -0.396 | 1.251 | Stablising | Unfavourable | 0.54 |
|  | T329M | -0.024 | 0.276 | Destablising | Favourable | 0.29 |
|  | A359S | -1.788 | -0.184 | Destablising | Favourable | -2.52 |
|  | T366I | 0.043 | 0.593 | Destablising | Unfavourable | 1.4 |
|  | P368S | -0.59 | -0.415 | Destablising | Unfavourable | -0.63 |
|  | A414S | -0.068 | -0.707 | Stablising | Favourable | -2.04 |
|  | P20S | -0.484 | 0.928 | Destablising | Favourable | -0.26 |
|  | D81Y | -0.451 | 0.372 | Destablising | Favourable | -0.14 |
|  | G178V | -0.558 | 0.481 | Destablising | Unfavourable | 0.15 |
|  | A208S | -0.567 | -0.357 | Stablising | Favourable | -1.75 |
|  | A305V | -0.01 | -0.272 | Stablising | Unfavourable | -0.97 |
|  | A398V | -0.629 | -0.516 | Stablising | Favourable | -0.97 |
|  | S79T | -0.307 | -0.114 | Stablising | Favourable | 0.16 |
|  | S188P | -0.328 | 1.185 | Stablising | Favourable | -0.1 |
|  | S327W | -1.154 | 0.522 | Destablising | Unfavourable | 1.3 |
